# Palovarotene for Fibrodysplasia Ossificans Progressiva (FOP): Results of a Randomized, Placebo‐Controlled, Double‐Blind Phase 2 Trial

**DOI:** 10.1002/jbmr.4655

**Published:** 2022-08-17

**Authors:** Robert J. Pignolo, Geneviève Baujat, Edward C. Hsiao, Richard Keen, Amy Wilson, Jeff Packman, Andrew L. Strahs, Donna R. Grogan, Frederick S. Kaplan

**Affiliations:** ^1^ Department of Medicine, Divisions of Geriatric Medicine and Gerontology, Hospital Internal Medicine, and Endocrinology, Robert and Arlene Kogod Center on Aging Mayo Clinic Rochester MN USA; ^2^ Departement de Genetique Institut IMAGINE and Hôpital Necker‐Enfants Malades Paris France; ^3^ Division of Endocrinology and Metabolism, UCSF Metabolic Bone Clinic, Institute for Human Genetics, Institute for Regeneration Medicine, and the Program for Craniofacial Biology University of California‐San Francisco San Francisco CA USA; ^4^ Consultant Rheumatologist & Honorary Senior Lecturer in Metabolic Bone Disease The Royal National Orthopaedic Hospital Stanmore UK; ^5^ Ipsen Newton MA USA; ^6^ Departments of Orthopaedic Surgery and Medicine, and The Center for Research in FOP and Related Disorders, Perelman School of Medicine University of Pennsylvania Philadelphia PA USA

**Keywords:** CELL/TISSUE SIGNALING, CLINICAL TRIALS, FIBRODYSPLASIA OSSIFICANS PROGRESSIVA, HETEROTOPIC OSSIFICATION, RADIOLOGY

## Abstract

Fibrodysplasia ossificans progressiva (FOP) is an ultra‐rare genetic disorder characterized by progressive heterotopic ossification (HO), often heralded by flare‐ups, leading to reduced movement and life expectancy. This placebo‐controlled, double‐blind trial (NCT02190747) evaluated palovarotene, an orally bioavailable selective retinoic acid receptor gamma agonist, for prevention of HO in patients with FOP. Patients experiencing a flare‐up were enrolled in two cohorts: (1) patients ≥15 years were randomized 3:1 to palovarotene 10/5 mg (weeks 1–2/3–6) or placebo; (2) patients ≥6 years were randomized 3:3:2 to palovarotene 10/5 mg, palovarotene 5/2.5 mg (weeks 1–2/3–6), or placebo. Cohort data were pooled. The primary endpoint was the proportion of responders (no/minimal new HO at flare‐up body region by plain radiograph) at week 6. Change from baseline in HO volume and new HO incidence were assessed by computed tomography (CT) at week 12. Tissue edema was assessed by magnetic resonance imaging (MRI) or ultrasound. Forty patients (aged 7–53 years) were enrolled (placebo: *n* = 10; palovarotene 5/2.5 mg: *n* = 9; palovarotene 10/5 mg: *n* = 21). Disease history was similar between groups. In the per‐protocol population, the proportion of responders at week 6 by plain radiograph was 100% with palovarotene 10/5 mg; 88.9% with palovarotene 5/2.5 mg; 88.9% with placebo (Cochran‐Armitage trend test: *p* = 0.17). At week 12, the proportions were 95.0% with palovarotene 10/5 mg; 88.9% with palovarotene 5/2.5 mg; 77.8% with placebo (Cochran‐Armitage trend test: *p* = 0.15). Week 12 least‐squares mean (LSmean) new HO volume, assessed by CT, was 3.8 × 10^3^ mm^3^ with palovarotene 10/5 mg; 1.3 × 10^3^ mm^3^ with palovarotene 5/2.5 mg; 18.0 × 10^3^ mm^3^ with placebo (pairwise tests versus placebo: *p* ≤ 0.12). Palovarotene was well‐tolerated. No patients discontinued treatment or required dose reduction; one patient had dose interruption due to elevated lipase. Although these findings were not statistically significant, they support further evaluation of palovarotene for prevention of HO in FOP in larger studies. © 2022 The Authors. *Journal of Bone and Mineral Research* published by Wiley Periodicals LLC on behalf of American Society for Bone and Mineral Research (ASBMR).

## Introduction

Fibrodysplasia ossificans progressiva (FOP; OMIM #135100) is an ultra‐rare genetic disorder with an estimated prevalence of up to 1.4 per million individuals.^(^ ^1^ ^)^ Beginning in childhood, the disease is characterized by painful, recurrent, episodic extraskeletal bone formation, known as heterotopic ossification (HO), often preceded by inflammatory soft tissue swelling, referred to as flare‐ups. Over time, HO results in progressive ankylosis of major joints with resultant loss of movement; many individuals with FOP are confined to a wheelchair by their 20s, requiring lifelong caregiver assistance to perform activities of daily living.^(^ ^2^, ^3^, ^4^ ^)^ The estimated median lifespan of individuals with FOP is 56 years;^(^ ^5^ ^)^ death is often due to cardiorespiratory failure (as a result of thoracic insufficiency syndrome which is usually accelerated by progressive restrictive chest wall disease).^(^ ^2^, ^5^ ^)^

FOP is caused by activating missense mutations in the activin A receptor type I gene (*ACVR1*; also known as activin‐like kinase 2 [*ALK2*]), which encodes a bone morphogenetic protein (BMP) type I receptor.^(^ ^6^ ^)^ Approximately 97% of patients with FOP have the same *ACVR1* ^R206H^ point mutation;^(^ ^7^ ^)^ the mechanism of disease is therefore specific and provides a target for drug development.^(^ ^8^ ^)^ However, there are currently no effective treatment options to prevent or block HO in FOP; current guidelines recommend the use of corticosteroids and nonsteroidal anti‐inflammatory drugs for palliative management.^(^ ^9^ ^)^

HO in FOP occurs through an endochondral pathway.^(^ ^10^ ^)^ Retinoid signaling is a strong inhibitor of chondrogenesis, a requisite step in endochondral ossification.^(^ ^11^ ^)^ The retinoid nuclear signaling pathway and nuclear retinoic acid receptors (RARs) play essential roles in regulating chondrogenic differentiation.^(^ ^12^, ^13^ ^)^ RARγ agonists potently impede heterotopic endochondral ossification by downregulation of BMP signaling in prechondrogenic cells by promoting the degradation of BMP‐pathway specific Smad 1/5/8 of the mutated *ALK2/ACVR1* gene, and by redirecting prechondrogenic mesenchymal stem cells from an osteoblast fate to a non‐osseous soft tissue fate.^(^ ^12^ ^)^ Palovarotene is an orally bioavailable, selective RARγ agonist originally developed for the treatment of chronic obstructive pulmonary disease (COPD),^(^ ^14^ ^)^ and is believed to act via this pathway to prevent HO in animal models of FOP.^(^ ^12^, ^15^, ^16^ ^)^ Across indications in the palovarotene clinical programme, over 1200 individuals have received at least one dose of palovarotene, including 309 healthy volunteers, 611 patients with COPD, 164 patients with FOP, and 129 patients with multiple osteochondromas.

Here, we present the results from a randomized, double‐blind, placebo‐controlled, phase 2, interventional, dose‐finding trial to evaluate the ability of different doses of palovarotene to prevent HO following flare‐ups in patients with FOP. The effect of palovarotene on flare‐up symptoms and patient‐reported outcomes (PROs) was also investigated.

## Patients and Methods

### Patients

Patients included were ≥6 years of age, clinically diagnosed with classic FOP, had flare‐up onset in an appendicular area, abdomen, or chest within 7 days prior to randomization, and were receiving current standard of care.^(^ ^9^ ^)^ Flare‐ups were defined by the presence of two or more patient‐reported symptoms of pain, soft tissue swelling, decreased range of motion, stiffness, redness, or warmth,^(^ ^17^ ^)^ and were required to be confirmed by the investigator and have a patient‐reported onset date. Sexually active patients were required to agree to remain abstinent or use two highly effective forms of birth control. Pregnancy testing was performed before and during treatment in females of child‐bearing potential. All patients gave signed and dated informed patient/parent consent or age‐appropriate assent (as per local regulations).

### Trial design

**Fig. 1:**
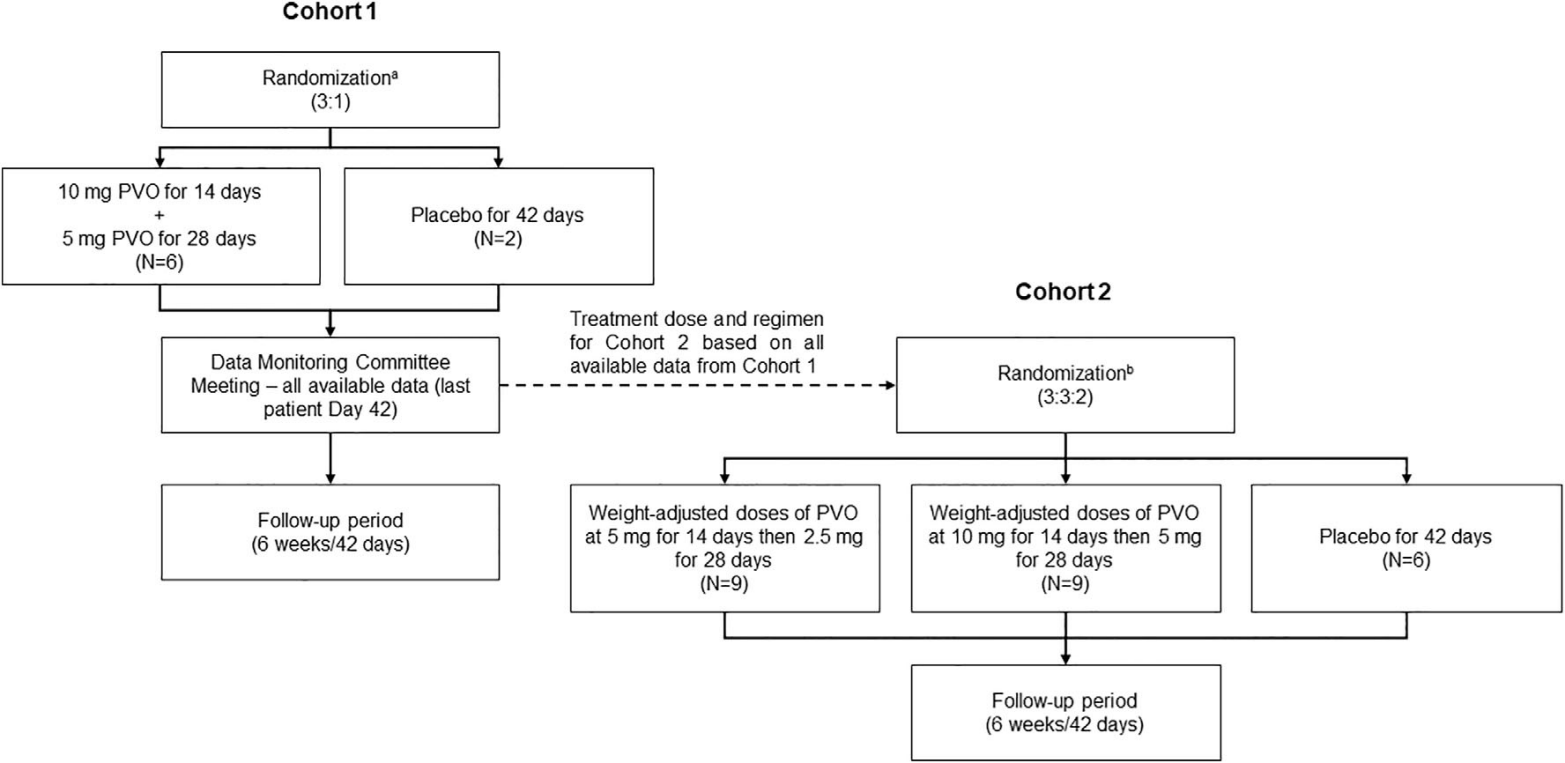
Schematic showing the randomization of patients in Cohorts 1 and 2. ^a^Includes eight patients with eligible flare‐ups randomized in a 3:1 ratio to either PVO 10/5 mg or placebo. ^b^Treatment and dosing regimens for Cohort 2 were based on the data available for Cohort 1. During the Data Monitoring Committee review, enrollment continued during the interim between randomization of the last patient in Cohort 1 and the start of Cohort 2 to allow eligible patients to participate; an additional eight patients with eligible flare‐ups were randomized in a 3:1 ratio to either PVO 10/5 mg or placebo. PVO = palovarotene.

Patients were enrolled in this phase 2, multicenter, randomized, double‐blind, sponsor‐unblinded, placebo‐controlled, dose‐ranging trial (Clinical Trials: https://clinicaltrials.gov/ct2/show/NCT02190747) within 7 days of flare‐up onset (day of first dose of study treatment was considered baseline). Enrollment was followed by a 6‐week treatment period, then a 6‐week follow‐up period (Fig. S1). Patients were randomized within two cohorts, each of which continued for the duration of the study (Fig. 1). For each cohort, a list of patient numbers and randomized treatment allocations was prepared and allocated via an interactive web response system; treatment allocations remained blinded. Cohort 1 was specified to include patients aged ≥15 years randomized 3:1 to oral palovarotene 10 mg daily for 2 weeks followed by 5 mg for 4 weeks (palovarotene 10/5 mg; weeks 1–2/3–6) or placebo for 6 weeks (weeks 1–6). A Data Monitoring Committee (DMC) review of unblinded safety and efficacy data from Cohort 1 recommended that the trial proceed as planned and determined appropriate dosing regimens for Cohort 2. In Cohort 2, patients aged ≥6 years were randomized 3:3:2 to palovarotene 10/5 mg, palovarotene 5 mg daily for 2 weeks followed by 2.5 mg for 4 weeks (palovarotene 5/2.5 mg; weeks 1–2/3–6), or placebo for 6 weeks; doses in Cohort 2 were weight‐adjusted as in Table S1.

The first patient was enrolled in July 2014 and the last patient completed the trial in May 2016. All study sites (United States: *n* = 2; France: *n* = 1; United Kingdom: *n* = 1) obtained approval from their local institutional review boards and complied with all applicable national, local, ethical, and regulatory guidelines.

### Imaging endpoints

The schedule of assessments is shown in Table S2. The primary efficacy endpoint was the proportion of responders in the anterior and lateral projections of the flare‐up body region assessed by plain radiograph at week 6. Responders were defined as those with no or minimal new HO at the flare‐up body region, as indicated by a HO score ≤3 on a scale from 0 to 6 (lower score indicates less HO; Table S3).^(^ ^18^ ^)^ Secondary endpoints included the same assessment at week 12, the volume of new HO at the flare‐up body region (assessed by low‐dose computed tomography [CT] scan) at weeks 6 and 12, and presence of soft tissue swelling/edema at the flare‐up body region (assessed by magnetic resonance imaging [MRI] or ultrasound in patients unable to undergo MRI).

Plain radiographs were initially chosen as an imaging modality due to ease of performance and limited radiation exposure, being the standard imaging procedure used in the clinical care of patients with FOP at the time of the study design and allowing scoring of HO as in Table S3. However, examination of both plain radiographs and CT scans demonstrated that CT scans were more sensitive in the quantification of the amount of new HO than plain radiographs. Thus, additional non‐prespecified analyses included the proportion of patients with any new HO at the flare‐up body region at weeks 6 and 12 as assessed by CT scans (or plain radiograph if CT scans were not available). First, qualitative assessment of scans was performed by readers to determine whether or not any new HO was present; no threshold values for voxel or pixel intensity were used. If new HO was deemed to be present at the flare‐up body region, volume of new HO was assessed by CT scan and area of new HO by plain radiograph.

Interpretations of radiographic, CT, MRI, and ultrasound images were performed at a central imaging laboratory using two prespecified procedures. In the primary read (performed by two independent treatment‐blinded musculoskeletal radiologists with consensus adjudication to agree on each final read result), the presence/absence, volume, and area of baseline HO and postbaseline new HO, and soft tissue edema were recorded on each relevant imaging modality at the flare‐up body region. Readers independently compared baseline and postbaseline images within a modality. Each imaging modality was assessed independently for HO and/or soft tissue edema; comparisons could not be made between modalities.

A global read was subsequently performed to determine the presence/absence and severity of edema and new HO at the flare‐up body region through full evaluation of all images across all modalities and time points in conjunction. Volume and area of new postbaseline HO were not recalculated in the global read. In the global read process, one of the central imaging laboratory musculoskeletal radiologists, an independent musculoskeletal consultant radiologist, an independent consultant ultrasound radiologist, and the investigators (all of whom were treatment‐blinded) met to read all scans across all modalities and time points, with consensus adjudication to agree on each final read result. This global read process was added to reduce potential variability created by the original, prospective primary read process.

For outcomes for which both a primary read and a global read were performed (presence/absence and severity of new HO and edema), global read values were prioritized for reporting as this more comprehensive process is believed to assess flare‐up outcomes more accurately. For other imaging outcomes (volume and area of new HO) where no global read was performed, primary read values are reported.

### Flare‐up symptoms and duration

Flare‐up duration was determined using a daily diary in which patients indicated “yes” or “no” in response to the question “Is your flare‐up still ongoing?”. Flare‐up resolution was defined as the day after the last “yes” response was recorded. Changes from baseline in patient‐reported pain and swelling at the flare‐up body region were assessed using numeric rating scales (NRS) at weeks 2, 4, 6, 9, and 12. Scores ranged from 0 (no pain or swelling) to 10 (worst pain or swelling ever experienced). For children <8 years of age, pain was assessed using the Faces Pain Scale‐Revised (FPS‐R), for which scores ranged from 0 (no pain) to 10 (very much pain).^(^ ^19^ ^)^

### Patient‐reported outcome measures

Changes from baseline in physical function and ability to perform activities of daily living were assessed using age‐appropriate FOP‐Physical Function Questionnaire (FOP‐PFQ) forms administered at weeks 2, 4, 6, 9, and 12.^(^ ^20^, ^21^, ^22^ ^)^ Scores were expressed as a percentage of the worst possible score; lower percentages indicated better functioning.

Changes from baseline in physical and mental health were assessed at weeks 2, 4, 6, 9, and 12 using the Patient‐Reported Outcome Measure Information System (PROMIS) Global Physical and Global Mental Health Scales for adults and the PROMIS Pediatric Global Health Scale (proxy‐ and/or self‐completed forms) for children.^(^ ^22^, ^23^, ^24^ ^)^ Scores were converted to *T*‐scores such that a value of 50 (standard deviation: 10) represented the average for the general population in the United States. Higher *T*‐scores indicated better physical/mental health.

### Safety endpoints

Safety was monitored from the time informed consent was signed through to week 12. Any adverse event (AE) reported after the first dose of study treatment, up to and including the week 12 visit, was considered treatment‐emergent. Serious AEs were defined as life‐threatening or leading to death, hospitalization or prolongation of hospitalization, persistent or significant disability/incapacity, congenital anomaly, or any other medically important event. AEs were coded using the Medical Dictionary for Regulatory Activities (MedDRA; version 17.0); the relatedness of the AE to study treatment was determined by the investigators. Other safety evaluations included the assessment of suicide ideation/behavior using the Columbia‐Suicide Severity Rating Scale (C‐SSRS),^(^ ^25^ ^)^ clinical safety laboratory findings, electrocardiograms (ECGs), and vital signs. Evaluation of patients <18 years of age enrolled with open growth plates included knee and hand/wrist radiographs, and linear growth assessed by stadiometer and knee height.

### Biomarkers

Blood and urine samples for cartilage, bone, angiogenesis, and inflammation biomarkers were obtained at baseline and weeks 2, 4, 6, and 12 (Table S4). Upper and lower limit of normal (ULN and LLN) values were defined according to central laboratory normal values.

### Sample size

The proportion of patients with no or minimal HO (primary endpoint) was hypothesized to be 20% with placebo and 80% with successful palovarotene treatment, based on patient survey data and nonclinical data, respectively.^(^ ^18^ ^)^ Assuming that a higher dose results in increased efficacy, the power to detect a significant trend over the dose range (Cochran‐Armitage test of trend; *α* = 0.05) was tested in six scenarios, based on hypothesized efficacy, with chosen sample sizes pooled across Cohort 1 and Cohort 2: placebo: *n* = 10; palovarotene 5/2.5 mg: *n* = 9; palovarotene 10/5 mg: *n* = 21 (Table S5).

### Statistical analysis

The safety population included all patients who received at least one dose of study treatment, the full analysis set included all patients who had at least one evaluable postbaseline plain radiograph or CT scan, and the per protocol population included all patients with ≥80% compliance with treatment. All efficacy results are reported for the per protocol population, except FOP‐PFQ and PROMIS scores, which are reported for the full analysis set. The primary endpoint and analyses of proportions of patients with new HO were assessed using the Cochran‐Armitage test of trend (one‐sided). Pairwise comparisons of volume of new HO were performed using a repeated measures mixed model. Changes from baseline in the mean volume of HO, NRS scores for pain and swelling, and FOP‐PFQ and PROMIS scores were analyzed with a repeated measures mixed model with treatment, visit, and interaction for treatment and visit as fixed effects, and baseline value as a covariate. Results were summarized using least‐squares mean (LSmean) estimates and standard errors (SEs) from the model. Raw means are presented for comparison in Table S7.

## Results

### Patient disposition

**Fig. 2:**
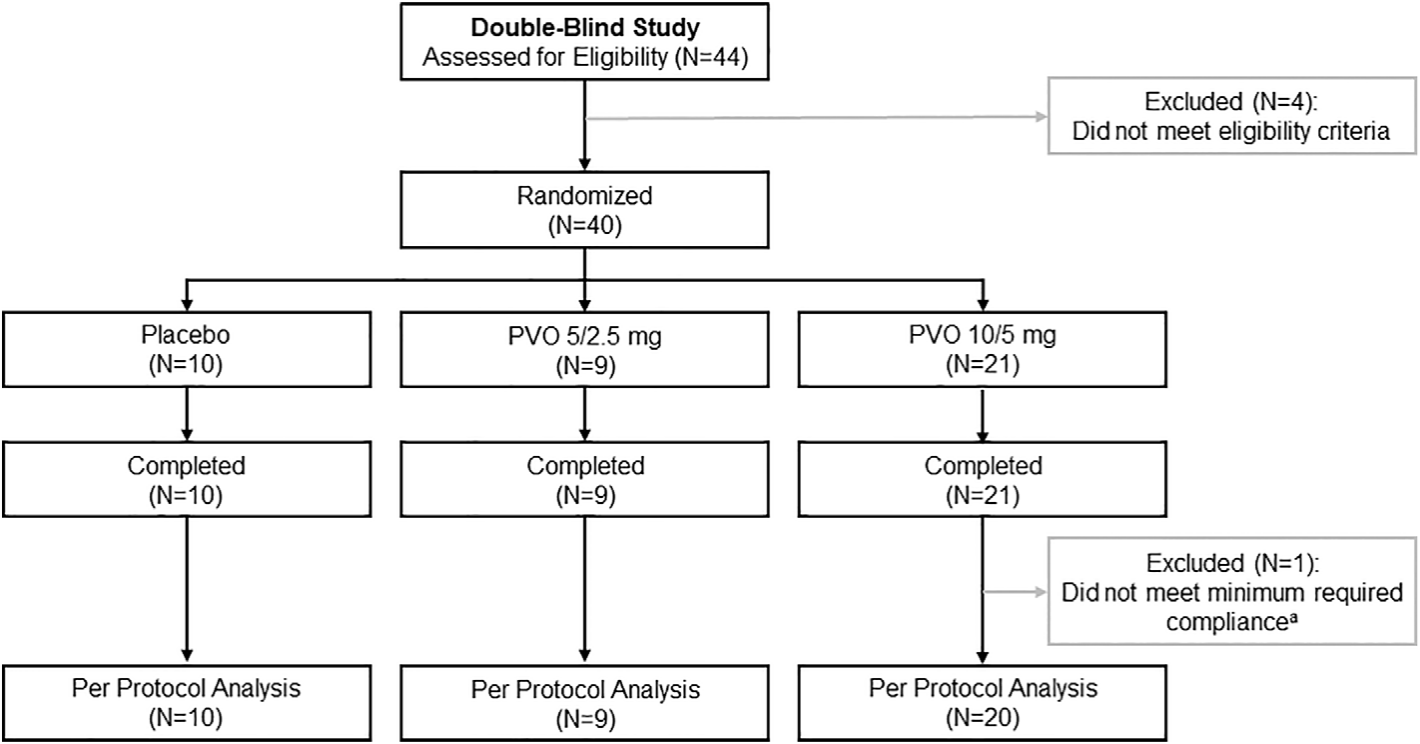
Patient disposition. ^a^Minimum required compliance with treatment: ≥80%; excluded patient compliance: 60%. PVO = palovarotene.

Eight patients were enrolled in Cohort 1 and 24 patients were enrolled in Cohort 2. Between randomization of the last patient in Cohort 1 and the start of Cohort 2, eight additional patients were randomized 3:1 to palovarotene 10/5 mg or placebo (Fig. 1). In total, 40 patients were enrolled (placebo: *n* = 10; palovarotene 5/2.5 mg: *n* = 9; palovarotene 10/5 mg: *n* = 21); all completed the trial and were enrolled in the open‐label extension (https://clinicaltrials.gov/ct2/show/NCT02279095; Fig. 2). One patient in the palovarotene 10/5 mg group was excluded from the per protocol analysis, for which efficacy outcomes are reported here, due to having only 60% treatment compliance; all other patients were included (overall treatment compliance: 98%).

### Baseline demographics and disease characteristics

Patient demographics and FOP disease history are summarized in Tables 1 and 2. Demographics were similar across all groups, with the exception of a smaller proportion of males in the placebo and palovarotene 5/2.5 mg groups compared with the palovarotene 10/5 mg group. Patients in all treatment groups had substantial HO at baseline (Table 2).

**Table 1:** Baseline Demographics by Treatment Group: Full Analysis Set

Parameter	Placebo (*n* = 10)	PVO 5/2.5 mg (*n* = 9)	PVO 10/5 mg (*n* = 21)
Age (years)			
Mean ± SD	21.2 ± 13.7	17.9 ± 8.6	22.8 ± 10.3
Median (range)	15.5 (9–53)	21.0 (7–29)	21.0 (9–44)
Patients 6–<15 years, *n* (%)	5 (50.0)	4 (44.4)	4 (19.0)
Sex, *n* (%)			
Male	3 (30.0)	3 (33.3)	12 (57.1)
Race, *n* (%)			
White	6 (85.7)	7 (77.8)	12 (80.0)
Black/African American	0	1 (11.1)	1 (6.7)
Asian	1 (14.3)	0	0
Multiple	0	1 (11.1)	2 (13.3)
Not available	3	0	6
Height (cm)			
*n*	8	9	19
Mean ± SD	153.4 ± 19.7	144.4 ± 19.2	160.9 ± 18.4
Median (range)	161.0 (127–175)	144.8 (121–166)	160.6 (130–191)
Weight (kg)			
*n*	9	9	21
Mean ± SD	48.2 ± 18.7	42.8 ± 16.1	58.1 ± 20.1
Median (range)	50.8 (22–73)	47.6 (20–64)	54.4 (28–108)

PVO = palovarotene; SD = standard deviation.

**Table 2:** Flare‐Up History and Attributes by Treatment Group: Full Analysis Set

Parameter	Placebo (*n* = 10)	PVO 5/2.5 mg (*n* = 9)	PVO 10/5 mg (*n* = 21)
**Flare‐Up History**			
Age at first confirmation of HO (years)			
Mean ± SD	4.1 ± 5.0	3.7 ± 3.6	6.2 ± 4.7
Median (range)	2.5 (0.3–17)	3.0 (0.3–12)	5.0 (1–17)
Months since last flare‐up prior to trial enrollment			
Mean ± SD	5.5 ± 4.5	18.7 ± 37.0	14.1 ± 25.7
Median (range)	4.3 (0.4–13)	2.5 (0.7–115)	4.8 (0.2–110)
Estimated flare‐ups per year			
*n*	10	9	20
Mean ± SD	2.3 ± 1.3	2.0 ± 1.9	4.6 ± 7.3
Median (range)	2.0 (1–5)	1.0 (0–6)	2.0 (1–30)
Number of anatomical sites with HO by physical examination			
Mean ± SD	12.3 ± 2.9	10.8 ± 4.4	11.4 ± 4.5
Median (range)	12.0 (8–19)	12.0 (1–16)	11.0 (3–19)
**Flare‐Up Characteristics at Trial Entry**			
Primary flare‐up location, *n* (%)			
Hip	5 (50.0)	2 (22.2)	9 (42.9)
Knee	2 (20.0)	3(33.3)	4 (19.0)
Abdomen	1 (10.0)	0	1 (4.8)
Distal upper extremities	1 (10.0)	0	1 (4.8)
Elbow	1 (10.0)	0	3 (14.3)
Chest	0	1 (11.1)	0
Shoulder	0	3 (33.3)	1 (4.8)
Distal lower extremities	0	0	2 (9.5)
Number of patient‐reported flare‐up symptoms			
Mean ± SD	4.1 ± 1.5	5.1 ± 2.0	4.5 ± 1.7
Median (range)	4.0 (2–7)	5.0 (2–8)	4.0 (2–8)
Most common patient‐reported symptoms, *n* (%)			
Pain	8 (80.0)	9 (100.0)	21 (100.0)
Swelling	8 (80.0)	6 (66.7)	14 (66.7)
Stiffness	6 (60.0)	6 (66.7)	18 (85.7)
Decreased ROM	6 (60.0)	6 (66.7)	13 (61.9)
Baseline flare‐up pain (measured by NRS)^a^ ^,^ ^b^			
Mean ± SD	4.6 ± 3.2	3.1 ± 2.9	4.8 ± 2.3
Median (range)	4.5 (0–10)	3.0 (0–9)	5.0 (1–10)
Baseline flare‐up swelling (measured by NRS)^a^			
Mean ± SD	3.7 ± 3.4	3.6 ± 2.6	3.0 ± 3.2
Median (range)	2.5 (0–10)	3.5 (0–7)	2.0 (0–10)
Baseline HO by CT scan at the flare‐up body region,^c^ *n* (%)			
Present	5 (50.0)	6 (66.7)	16 (84.2)
Not present	5 (50.0)	3 (33.3)	3 (15.8)
Not evaluable/not done^d^	0	0	2
Using glucocorticoids for flare‐up,^e^ *n* (%)	9 (90.0)	7 (77.8)	20 (95.2)
Baseline edema by MRI/ultrasound at flare‐up body region,^c^ *n* (%)			
Present	6 (75.0)	6 (85.7)	9 (64.3)
Not present	2 (25.0)	1 (14.3)	5 (35.7)
Not evaluable/not done^d^	2	2	7
Plain radiograph availability at baseline, *n* (%)			
Yes	10 (100.0)	8 (88.9)	21 (100.0)
Yes but not evaluable	0	1 (11.1)	0
No	0	0	0
CT scan availability at baseline, *n* (%)			
Yes	9 (90.0)	9 (100.0)	20 (95.2)
Yes but not evaluable	1 (10.0)	0	0
No	0	0	1 (4.8)

CT = computed tomography; HO = heterotopic ossification; MRI = magnetic resonance imaging; NRS = numeric rating scale; PVO = palovarotene; ROM = range of motion; SD = standard deviation.

^a^ Pain and swelling were based on NRS scores ranging from 0 to 10, where 0 = no pain/swelling and 10 = worst pain/swelling ever experienced.

^b^ Pain was rated using the Faces Pain Scale‐Revised based on a scale of 0 to 10, where 0 = no pain and 10 = very much pain for children under 8 years of age.

^c^ Based on global read assessment of evaluable images.

^d^ “Not evaluable” indicates that imaging was performed but determination of outcome was not possible; “not done” indicates that imaging was not performed.

^e^ Start date within 9 days after the flare‐up start date.

Overall, patients began treatment (placebo or palovarotene) an average of 6 days (range: 3–8 days) after the start of their current flare‐up. The distribution of flare‐up sites at trial entry was similar between patients in the placebo and palovarotene 10/5 mg groups, both of which had more hip flare‐ups than the palovarotene 5/2.5 mg group (Table 2). On average, patients reported between four and five flare‐up symptoms; the most common were pain, stiffness, and swelling. Baseline HO and soft tissue edema (both assessed by imaging) were present at the flare‐up body region in most patients (HO: 50.0%–84.2%; edema: 64.3%–85.7%); nearly all patients received glucocorticoids to manage flare‐up symptoms (77.8%–95.2%).

### Imaging endpoints—new heterotopic ossification

#### Primary endpoint

In the primary read, the proportion of responders at week 6 was 88.9% with placebo (*n* = 8/9; *n* = 1 not evaluable), 88.9% with palovarotene 5/2.5 mg (*n* = 8/9), and 100% with palovarotene 10/5 mg (*n* = 20/20) as determined by plain radiograph, with no significant dose trend (one‐sided Cochran‐Armitage test of trend: *p* = 0.17).

#### Secondary analyses

In the primary read, at week 12 the proportion of responders was 77.8% with placebo (*n* = 7/9; *n* = 1 not evaluable), 88.9% with palovarotene 5/2.5 mg (*n* = 8/9), and 95.0% with palovarotene 10/5 mg (*n* = 19/20) as determined by plain radiograph; there was no significant dose trend (one‐sided Cochran‐Armitage test of trend: *p* = 0.15).

**Fig. 3:**
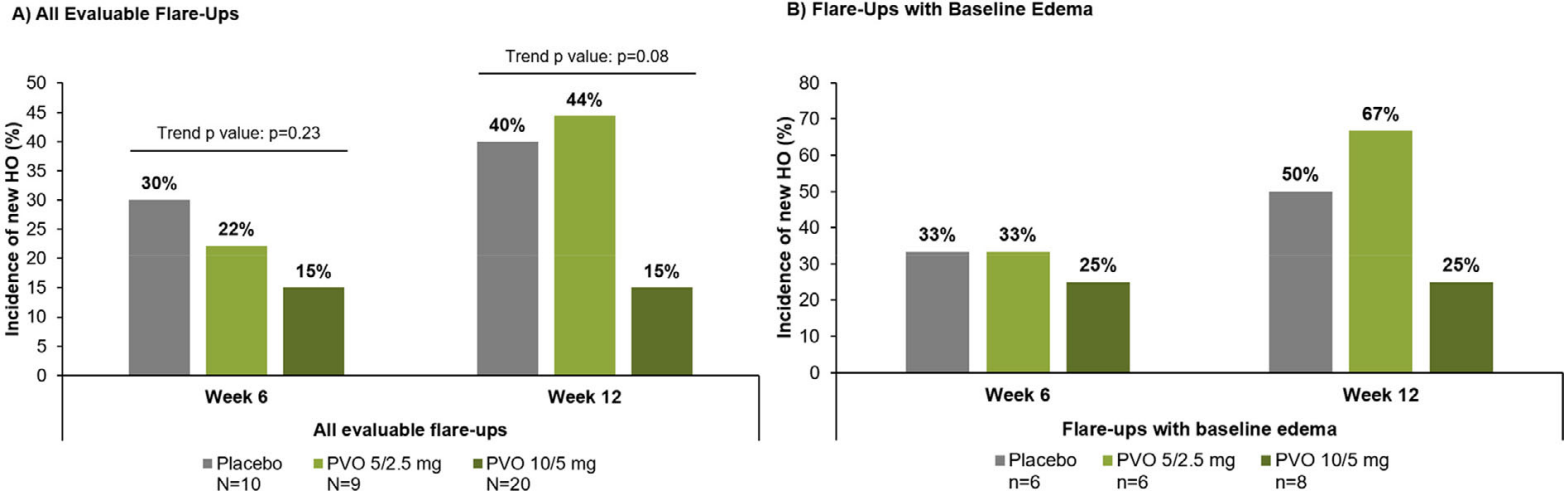
Incidence of new HO at the flare‐up body region at week 6 and week 12 as assessed by CT scan and/or plain radiograph (global read). (*A*) All evaluable flare‐ups. (*B*) Flare‐ups with baseline edema. Per protocol analysis set. In the global read, the presence/absence of new HO was determined after full evaluation of all images across all modalities and time points. (*A*) Primary analysis: one‐sided Cochran‐Armitage test of trend for all evaluable flare‐ups at week 6 and 12. CT = computed tomography; HO = heterotopic ossification; PVO = palovarotene.

In the global read, the proportion of flare‐ups with new HO at the flare‐up body region at week 12 (as assessed by CT scan and/or plain radiographs) was numerically lower in the palovarotene 10/5 mg group (*n* = 3/20; 15.0%) than in the placebo group (*n* = 4/10; 40.0%). The incidence of new HO at the flare‐up body region in the palovarotene 5/2.5 mg group (*n* = 4/9; 44.4%) was similar to placebo; there was no significant dose trend (Cochran‐Armitage test of trend: *p* = 0.08; Fig. 3A). Results were similar in the primary read (Fig. S2).

In the global read, baseline edema as assessed by MRI or ultrasound was present at the flare‐up body region in *n* = 6/8 in the placebo group (75.0%; *n* = 2 not evaluable), *n* = 6/7 in the palovarotene 5/2.5 mg group (85.7%; *n* = 2 not evaluable), and *n* = 8/13 in the palovarotene 10/5 mg group (61.5%; *n* = 7 not evaluable). Overall, the proportion of patients with new HO at the flare‐up body region at week 12 was numerically higher in those with flare‐ups with baseline edema versus those with no edema, and the proportion of patients with new HO at week 12 was lowest in the palovarotene 10/5 mg group (not tested for significance; Fig. 3B).

**Fig. 4:**
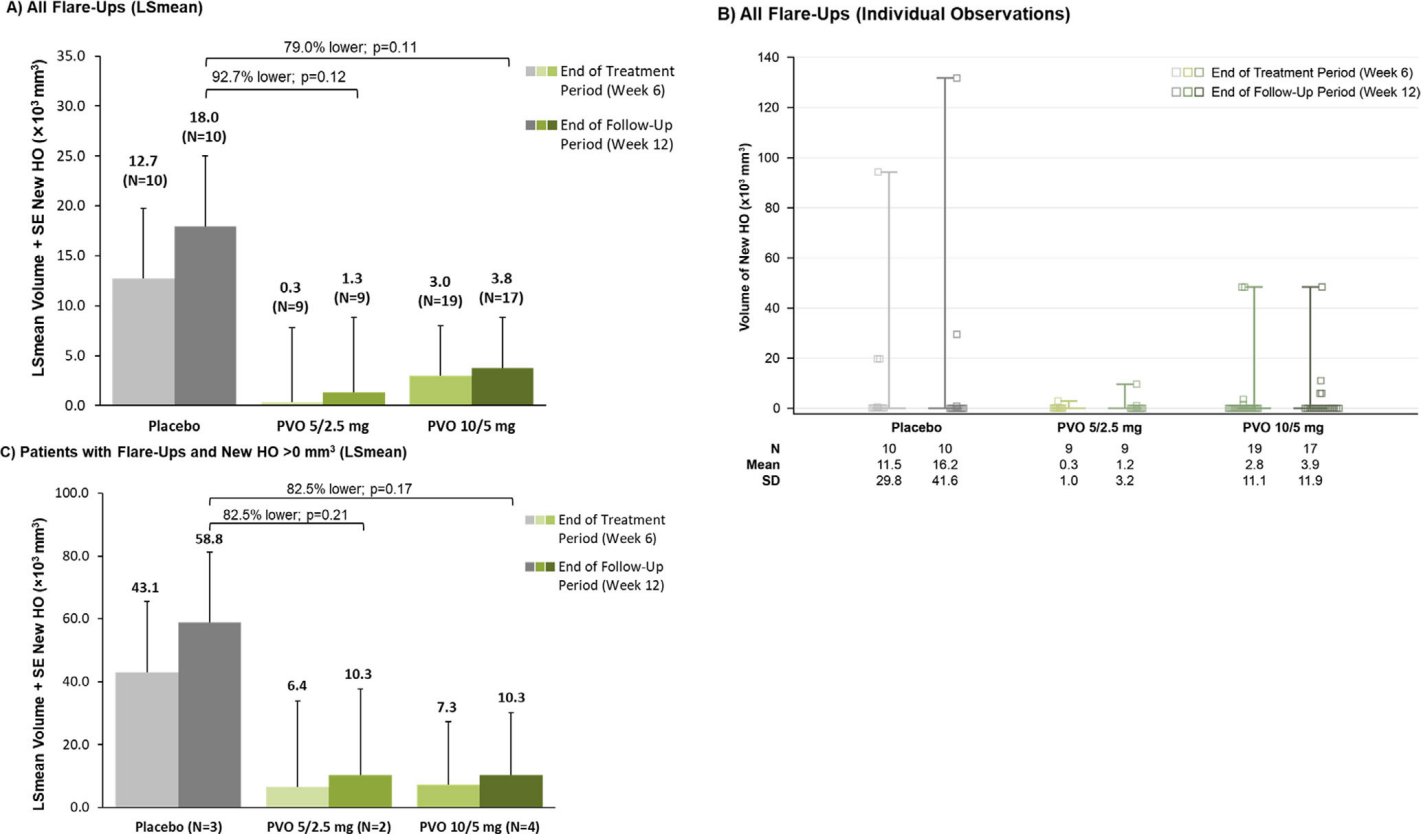
Volume of new HO at the flare‐up body region as measured by CT scan (primary read). (*A*) All flare‐ups (LSmean). (*B*) All flare‐ups (individual observations). (*C*) Patients with flare‐ups and new HO >0 mm^3^ (LSmean). Per protocol analysis set. In the primary read, each imaging modality was assessed independently for HO. *p* values are from pairwise tests from a repeated measures mixed model. (*A*) The volume of new HO was not evaluable in one patient at week 6 and three patients week 12, all in the PVO 10/5 mg group. (*B*) Squares represent individual observations. Whiskers extend to maximum and minimum values due to small interquartile ranges of data. (*C*) Flare‐up body regions: placebo: 2 hip, 1 knee; PVO 5/2.5 mg: 1 shoulder/elbow, 1 knee; PVO 10/5 mg: 3 hip, 1 knee. CT = computed tomography; HO = heterotopic ossification; LSmean = least squares mean; PVO = palovarotene; SD = standard deviation; SE = standard error.

Volume and area of new HO at the flare‐up body region were assessed only in the primary read. Using low‐dose CT scans, LSmean volume of new HO among all patients at week 12 was 92.7% lower with palovarotene 5/2.5 mg versus placebo and 79.0% lower with palovarotene 10/5 mg versus placebo; however, these differences were not significant (pairwise tests from a repeated measures mixed model: *p* = 0.12 and *p* = 0.11, respectively; Fig. 4*A*). Individual observations are shown in Fig. 4*B*. Similar differences were seen in the LSmean area of new HO at week 12 in both palovarotene groups versus placebo (Fig. S3). Among only those patients with new HO, the LSmean volume of new HO at week 12 was 82.5% lower in both palovarotene groups versus placebo; these differences were not significant (Fig. 4*C*).

### Flare‐up symptoms and duration

Patients receiving palovarotene 10/5 mg or palovarotene 5/2.5 mg reported shorter median (range) flare‐up duration versus placebo (22.0 [0–84] days or 28.0 [5–84] days versus 78.5 [14–84] days). Incidence of flare‐up resolution at week 12 was 82.4% (14/17 flare‐ups resolved; *n* = 3 not evaluable) with palovarotene 10/5 mg, 55.6% (5/9) with palovarotene 5/2.5 mg, and 62.5% (5/8; *n* = 2 not evaluable) with placebo. Median (range) duration of patient‐reported active symptoms was 13.0 (0–66) days with palovarotene 10/5 mg, 25.0 (5–84) days with palovarotene 5/2.5 mg, and 21.5 (2–84) days with placebo (not tested for statistical significance).

All groups, including placebo, had similar statistically significant decreases from baseline in flare‐up pain and swelling at week 12 (all *p* values ≤0.0016 from a repeated measures mixed model). The LSmean (SE) change from baseline in flare‐up pain at week 12 was −2.1 (0.7) with placebo, −2.6 (0.7) with palovarotene 5/2.5 mg, and −3.3 (0.5) with palovarotene 10/5 mg; the LSmean change from baseline in flare‐up swelling was −2.1 (0.7), −2.3 (0.7), and −2.2 (0.5), respectively; differences between palovarotene and placebo were not statistically significant using a repeated measures mixed model, although change from baseline in flare‐up pain was numerically greater with palovarotene 10/5 mg versus palovarotene 5/2.5 mg and placebo (Fig. S4).

### Patient‐reported outcome measures

Baseline FOP‐PFQ scores were similar across groups at baseline, as were the LSmean (SE) changes from baseline at week 12 (placebo: 3.0 [2.4]; palovarotene 5/2.5 mg: 1.1 [2.6]; palovarotene 10/5 mg: 4.1 [1.7]). Differences between placebo and the palovarotene groups were not significant at any assessed time point. Findings were similar in all groups for the PROMIS Global Health Scales for adults and children (Table S6).

### Biomarkers

Biomarker analysis found that, in general, most serum and urine biomarkers were within normal ranges in all treatment groups at baseline and all postbaseline time points (Table S4).

### Safety endpoints

All patients received at least one dose of study treatment. Mean exposure to palovarotene was 41.5 days (range: 38–42 days), with a mean total dose of 210.9 mg (range: 84–280 mg); exposure to placebo was similar (mean: 42.0 days; range: 40–44 days). All patients reported at least one treatment‐emergent AE; four patients reported serious AEs, and no AE led to discontinuation of the study treatment (Table 3). There were dose‐related increases in events typically associated with the use of retinoids, including dry skin, dry lips, pruritus, erythema, dermatitis acneiform, and dry mouth.

**Table 3:** Summary of Adverse Events: Safety Population

Parameter	Placebo (*n* = 10)	PVO 5/2.5 mg (*n* = 9)	PVO 10/5 mg (*n* = 21)
**Overview of treatment‐emergent AEs**, ***n*** **(** **%** **)**			
At least one AE^a^	10 (100.0)	9 (100.0)	21 (100.0)
Severe AEs	1 (10.0)	0	1 (4.8)
Serious AEs	1 (10.0)	1 (11.1)	2 (9.5)
AEs leading to discontinuation of study treatment	0	0	0
**Most common AEs,** ^b^ ***n*** **(%** **)**			
Skin and subcutaneous tissue disorders	4 (40.0)	6 (66.7)	19 (90.5)
Dry skin	3 (30.0)	5 (55.6)	17 (81.0)
Erythema	0	2 (22.2)	3 (14.3)
Pruritus generalized	0	1 (11.1)	4 (19.0)
Dermatitis acneiform	0	0	4 (19.0)
Pruritus	0	0	4 (19.0)
Gastrointestinal disorders	6 (60.0)	6 (66.7)	16 (76.2)
Lip dry	1 (10.0)	5 (55.6)	7 (33.3)
Nausea	2 (20.0)	1 (11.1)	6 (28.6)
Chapped lips	2 (20.0)	0	5 (23.8)
Diarrhea	1 (10.0)	0	4 (19.0)
Dry mouth	0	1 (11.1)	3 (14.3)
Musculoskeletal and connective tissue disorders	7 (70.0)	5 (55.6)	14 (66.7)
Arthralgia	6 (60.0)	1 (11.1)	10 (47.6)
Pain in extremity	2 (20.0)	3 (33.3)	2 (9.5)
General disorders and administration site conditions	3 (30.0)	4 (44.4)	14 (66.7)
Condition aggravated^c^	3 (30.0)	2 (22.2)	13 (61.9)
Pyrexia	1 (10.0)	3 (33.3)	1 (4.8)
Nervous system disorders	5 (50.0)	4 (44.4)	10 (47.6)
Headache	3 (30.0)	1 (11.1)	8 (38.1)

AE = adverse event; MedDRA = Medical Dictionary for Regulatory Activities; PVO = palovarotene.

^a^ Events that started after the first dose of study drug up to and including the last day of the study.

^b^ AEs that occurred in ≥3 patients in any group.

^c^ Intercurrent flare‐ups that occurred during the trial period, other than those that qualified the patient for enrollment, were recorded as AEs and coded to the preferred term “condition aggravated” using MedDRA version 17.0.

The AE “condition aggravated” (preferred term for intercurrent FOP flare‐ups) was reported more frequently by patients in the palovarotene 10/5 mg group than in the other groups (Table 3). Further examination did not reveal a consistent pattern of onset relative to palovarotene dosing (ie, these did not appear to be “rebound” flare‐ups that could potentially arise upon discontinuation of palovarotene treatment but were evenly distributed between the treatment and follow‐up periods).

Four patients had serious AEs: asthmatic crisis (one patient [placebo]; not related); hemorrhagic ovarian cyst (one patient [palovarotene 5/2.5 mg]; not related); condition aggravated (two patients [palovarotene 10/5 mg]; possibly related); four instances of myoclonus (all in the same patient [palovarotene 10/5 mg]; one event possibly related, three not related). The patient with multiple myoclonus events had a history of recurrent myoclonus episodes requiring hospitalization prior to enrollment.^(^ ^26^ ^)^ No patients required dose reductions. One patient receiving palovarotene 10/5 mg interrupted treatment due to transient, asymptomatic mild increased lipase (probably related). One patient receiving placebo interrupted treatment due to chest pain, pyrexia, and myalgias.

No patients reported Type 4 (active suicidal ideation with some intent to act, without specific plan) or Type 5 (active suicidal ideation with specific plan and intent) suicidal ideation using the C‐SSRS. There were few clinically relevant laboratory abnormalities. Elevations in lipase were intermittently observed in all groups, but most were also observed at screening (prior to study treatment administration) and at the end of the 6‐week follow‐up period at week 12 (off‐treatment). The elevations were often coincident with the use of high‐dose prednisone, were asymptomatic, and occurred intermittently throughout the 6‐week treatment period with the highest elevation observed in a patient receiving placebo.

There were no clinically relevant changes in ECG parameters or vital signs in any group. Of note, high percentages of patients in all treatment groups had conduction (*n* = 14/40; 35.0%) and/or rhythm abnormalities (*n* = 5/40; 12.5%) at baseline. Nonetheless, few patients had potentially clinically significant new‐onset abnormalities at the end of treatment at week 6: two palovarotene‐treated patients in the 10/5 mg‐treated group had high PR interval (>200 ms and/or increase ≥20 ms), and one patient from each treatment group had high QRS interval (>100 ms and/or increase ≥10 ms) on their ECGs.

Hand/wrist and knee radiographs were obtained in patients aged <18 years, with follow‐up performed in those who were <90% skeletally mature (defined as a bone age of 12 years for girls and 14 years for boys). At baseline, *n* = 9/18 (50%) evaluated had at least one epiphyseal bone abnormality; the most common was the presence of dense metaphyseal lines (39%). Additional baseline bone abnormalities observed in individual patients included sclerosis, undermineralization or osteopenia, and dysplasia. There were two postbaseline bone abnormalities observed during the 12‐week trial period, both in the palovarotene 5/2.5 mg group: one patient had dense metaphyseal lines in the knee that worsened from baseline, and one patient had sclerosis in the wrist that was not present at baseline. Overall, during this trial there were no apparent treatment‐related effects on growth plates, or linear or knee height (though highly variable in all groups).

## Discussion

There are no approved treatments for the prevention of HO in patients with FOP. This was the first phase 2 placebo‐controlled trial in FOP to investigate an episodic treatment to potentially prevent HO after a confirmed flare‐up. Although inconclusive, the data presented here suggest that high‐dose RARγ agonist treatment during a flare‐up may be beneficial in patients with FOP. Based on these data, palovarotene was advanced in the FOP clinical program.

Although there was no significant trend associated with palovarotene dose and the proportion of responders (HO score ≤3 on a scale from 0 to 6, where a lower score indicates less HO; ie, patients achieving no/minimal new HO at the flare‐up body region) as assessed by plain radiograph at week 6, the high proportion of responders within the placebo group suggests this imaging modality was not sufficiently sensitive to measure the presence or amount of new HO at the flare‐up body region. Indeed, CT scans were found to be more sensitive. In addition, the global read review process demonstrated the importance of reviewing across all available imaging modalities to determine the presence and extent of HO. Global read assessments of CT images and/or plain radiographs determined that the incidence of flare‐ups with new HO at the flare‐up body region at week 12 was numerically 63% lower with palovarotene 10/5 mg than with placebo, with no difference reported between palovarotene 5/2.5 mg and placebo. Results were similar with the primary read process.

The higher‐than‐expected proportion of responders in the placebo group may indicate that 6 weeks was not a long enough period to measure response. More than 50% of all patient‐reported flare‐ups in the trial did not result in new HO at the flare‐up body region, indicating either the presence of abortive flare‐ups, or that the use of patient survey data, in which patients self‐assessed loss of movement, led to an overestimate of the proportion of untreated flare‐ups that had new HO among those that caused loss of function or movement.^(^ ^17^ ^)^ The loss of movement reported in the survey may have instead been due to other factors unrelated to HO, such as severe developmental arthropathy.^(^ ^27^ ^)^ Use of prospective, objective data collection instead of survey data may improve estimates.

Categorical analysis of new HO is a stringent method for the assessment of potential treatment efficacy. In nonclinical pharmacology studies utilizing a continuous analysis, palovarotene dose‐related decreases in HO volume were observed.^(^ ^12^ ^)^ In the trial reported here, lower (although not statistically different) volumes of new HO at week 6 and week 12 were observed at the flare‐up body region with both palovarotene doses versus placebo. However, the mean volume of new HO increased at the flare‐up body region in both palovarotene groups in the 6 weeks following treatment cessation. This observation indicated that palovarotene could interfere with the process of new heterotopic bone formation, as seen in preclinical mouse studies,^(^ ^15^ ^)^ and that treatment would be required for as long as the HO process continued, suggesting outcomes may be optimized by increasing dose and treatment duration. These results informed decisions for palovarotene dosing during the open‐label extension of this placebo‐controlled trial (NCT02279095).^(^ ^28^ ^)^

Although the volume of new HO at weeks 6 and 12 was similar in the palovarotene 5/2.5 mg and palovarotene 10/5 mg groups, these results were likely influenced by flare‐up location. For example, hip flare‐ups are considered to be among the most long‐lasting and functionally disabling flare‐ups experienced by patients with FOP,^(^ ^18^ ^)^ and may be accompanied by large amounts of HO; three of the four flare‐ups that were associated with new HO in the palovarotene 10/5 mg group were located at the hip, compared with none in the palovarotene 5/2.5 mg group and two of three in the placebo group.

There were no significant differences in patient‐reported outcomes between the placebo and palovarotene treatment groups at any time point. However, this trial showed that significant progression of disease is difficult to detect over the relatively brief 12‐week time period considered here, consistent with data from an FOP Natural History Study (NHS).^(^ ^22^ ^)^ Rather, demonstrable progression in functional impairment due to HO accumulation would be expected over years;^(^ ^22^ ^)^ cumulative analogue joint involvement scale (CAJIS) scores, for example, are estimated to increase by only 0.5 points per year (as measured on a 0–30 scale).^(^ ^29^ ^)^

In this trial, the safety profile of palovarotene was similar to other retinoids.^(^ ^30^, ^31^, ^32^ ^)^ There were no deaths and no clinically relevant treatment‐related effects observed using clinical safety laboratory findings, vital signs, ECGs (for which findings were consistent with those from the FOP NHS^(^ ^33^ ^)^), growth plates, or linear height. The duration of this study was likely too short to fully assess growth plate changes; however, growth plate safety events have been closely monitored in subsequent studies that utilized higher doses of palovarotene for a longer period of time and premature growth plate closure and epiphyseal disorder events have been reported in the phase 3 MOVE trial (https://clinicaltrials.gov/ct2/show/NCT03312634).^(^ ^34^ ^)^ One non–retinoid‐associated finding was the higher incidence of intercurrent FOP flare‐ups (AE preferred term: condition aggravated) in the palovarotene 10/5 mg group relative to placebo. Review of these events did not reveal a consistent pattern of onset relative to palovarotene dosing, suggesting that these were not rebound flare‐ups following palovarotene discontinuation. In addition, all of these events were mild or moderate, and approximately one‐half were either related to trauma, observed at the same location as the qualifying flare‐up, or occurred during the follow‐up period only. Intercurrent flare‐ups are being carefully monitored in ongoing palovarotene clinical studies.

There are at least five possible reasons why this trial did not reach clinical significance. The lack of knowledge surrounding the natural history of flare‐up outcomes at the time this trial was designed led to it being underpowered. Findings from an FOP NHS were therefore utilized in the design of the phase 3 MOVE trial of palovarotene in FOP.^(^ ^22^ ^)^ Additionally, the originally planned detection methods were not sensitive enough. The calculation of the sample size required to detect a significant trend in the primary endpoint was based on the assumption that 20% of untreated flare‐ups would result in a HO score ≤3 on a scale from 0 to 6 (indicating no or minimal new HO) as assessed by plain radiographs, based on a survey of FOP patients with regard to flare‐ups,^(^ ^17^ ^)^ rather than the 90% reported in the placebo group here. This insensitive modality reduced the power of the trial and likely hampered the detection of a significant dose trend for the reduction of incidence and/or volume of new HO at flare‐up body regions; results were closer to those expected when the more sensitive CT imaging modality was used. These findings are important for the design of future trials in FOP, and have been taken into account in expert advice describing special considerations for clinical trials in patients with this disease.^(^ ^35^ ^)^ Another possible reason why this trial did not reach clinical significance was the 12‐week period over which it was conducted; this may have been too brief for new HO to be observed. Additionally, starting palovarotene an average of 6 days after flare‐up onset in this trial may have limited the potential for early efficacy of treatment. The final possible reason why this trial may not have reached clinical significance could be that palovarotene is ineffective at preventing new HO at the site of a flare‐up. However, this does not appear to be the case; although statistical significance was not reached as a result of the factors mentioned, the data clearly suggest a nonstatistically significant trend showing that treatment with palovarotene 10/5 mg resulted in lower new HO at the flare‐up body region compared with placebo (Figs. 3 and 4).

As with many clinical trials in ultra‐rare diseases, other limitations include the low number of patients enrolled. Baseline characteristics across the three groups were generally similar, however, the palovarotene 10/5 mg and placebo groups had higher mean age than the palovarotene 5/2.5 mg group; this was to be expected given that the inclusion criteria were different for Cohorts 1 and 2, and the palovarotene 5/2.5 mg dose arm was only included in Cohort 2, which had a lower age cutoff. Additionally, the proportion of patients with flare‐up edema at baseline was greater in the palovarotene 5/2.5 mg and placebo groups, though MRI/ultrasound scans were unavailable in a greater proportion of patients in the palovarotene 10/5 mg group, and the proportion of patients with HO at the flare‐up site at baseline was highest in the palovarotene 10/5 mg group (Table 2). Another potential limitation to be considered is that the threshold for self‐reporting of a flare‐up was subtly influenced by the design of this first‐in‐human interventional trial, in which the self‐reporting of a flare‐up would trigger treatment administration.

In conclusion, when administered for 6 weeks at the start of a flare‐up and compared with placebo, palovarotene dosed at 10/5 mg resulted in a lower proportion of patients with FOP experiencing new HO, and a lower volume of new HO at the flare‐up body region. Although these findings did not achieve statistical significance versus placebo, a combination of chronic and flare‐up palovarotene dosing regimens has shown promising trends in the ongoing open‐label extension of this trial (NCT02279095) and the phase 3 MOVE trial (NCT03312634).^(^ ^34^, ^36^ ^)^ Furthermore, palovarotene was well‐tolerated and primarily associated with the occurrence of mucocutaneous AEs. These findings support further evaluation of palovarotene as a potential treatment for preventing HO in patients with FOP.

## AUTHOR CONTRIBUTIONS

**Robert Pignolo:** Conceptualization; investigation; visualization; writing – review and editing. **Geneviève Baujat:** Conceptualization; investigation; visualization; writing – review and editing. **Edward Hsiao:** Conceptualization; investigation; visualization; writing – review and editing. **Richard Keen:** Conceptualization; investigation; visualization; writing – review and editing. **Amy Wilson:** Conceptualization; visualization; writing – review and editing. **Jeff Packman:** Conceptualization; project administration; visualization; writing – review and editing. **Andrew L. Strahs:** Conceptualization; data curation; formal analysis; methodology; visualization; writing – review and editing. **Donna R. Grogan:** Conceptualization; methodology; project administration; visualization; writing – review and editing. **Frederick Kaplan:** Conceptualization; investigation; visualization; writing – review and editing.

## Conflicts of Interest

RJP: Research investigator: Clementia/Ipsen, Regeneron; Advisory board: International FOP Association (IFOPA) Medical Advisory Board; President of the International Clinical Council on FOP. GB: Advisory board: Clementia/Ipsen, FOP European Consortium, International Clinical Council on FOP; Speaker: Clementia/Ipsen; ECH: Research investigator: Clementia/Ipsen; Receives clinical trials support through his institution from Clementia/Ipsen; Prior research support from Neurocrine Biosciences and Regeneron; Serves in a volunteer capacity on the International Clinical Council on FOP, the medical advisory board of the Fibrous Dysplasia Foundation, and the medical registry advisory board of the IFOPA. RK: Research investigator: Ipsen/Clementia, Kyowa Kirin, Regeneron; Advisory board: IFOPA FOP Registry Medical Advisory Board, International Clinical Council on FOP. AW: Employee of Clementia at the time the study was conducted. JP: Employee of Clementia at the time the study was conducted. ALS: Employee of Ipsen. DRG: Chief Medical Officer of Clementia and a shareholder at the time these data were obtained. FSK: Research investigator: Clementia/Ipsen, Regeneron; Advisory Board: IFOPA Medical Advisory Board; Founder and Past President of the International Clinical Council on FOP. In April 2019, Ipsen acquired Clementia Pharmaceuticals.

### PEER REVIEW

The peer review history for this article is available at https://publons.com/publon/10.1002/jbmr.4655.

## Supporting information


**Table S1** Weight‐Adjusted Palovarotene Doses
**Table S2**. Schedule of Key Assessments at Baseline and Weeks 6 and 12
**Table S3**. Heterotopic Ossification Scores (from Rajapakse and colleagues^(19)^)
**Table S4**. Incidence of Biomarker Readings Above the Upper Limit of Normal or Below the Lower Limit of Normal by Treatment Group
**Table S5**. Power to Detect a Significant Dose Trend Under Various Assumptions of Response Across the Three Treatment Groups
**Table S6**. LSmean Change from Baseline Scores for FOP‐PFQ and PROMIS at Week 12
**Table S7**. Raw Means and LSmeans for New HO Volume, New HO Area, Pain and Swelling NRS, and Changes from Baseline in FOP‐PFQ and Adult and Pediatric PROMIS at Week 12
**Fig. S1**. Trial Schematic
**Fig. S2**. Incidence of Flare‐Ups with New HO at the Flare‐Up Body Region at Week 6 and Week 12 as Assessed by CT Scan or Plain Radiograph (Primary Read)
**Fig. S3**: LSmean Area of New HO at the Flare‐Up Body Region in All Flare‐Ups as Assessed by Plain Radiograph (Primary Read)
**Fig. S4**. LSmean Change from Baseline in Pain Numeric Rating Scale at Each Assessed Time Point

## Data Availability

Where patient data can be anonymized, Ipsen will share all individual participant data that underlie the results reported in this article with qualified researchers who provide a valid research question. Study documents, such as the study protocol and clinical study report, are not always available. Proposals should be submitted to DataSharing@Ipsen.com and will be assessed by a scientific review board. Data are available beginning 6 months and ending 5 years after publication; after this time, only raw data may be available.

## References

[1] Liljesthröm M , Pignolo R , Kaplan F . Epidemiology of the global fibrodysplasia ossificans progressiva (FOP) community. J Rare Dis Res Treat. 2020;5:31‐36.

[2] Pignolo RJ , Shore EM , Kaplan FS . Fibrodysplasia ossificans progressiva: diagnosis, management, and therapeutic horizons. Pediatr Endocrinol Rev. 2013;10(Suppl 2):437‐448. 23858627 PMC3995352

[3] Ortiz‐Agapito F , Colmenares‐Bonilla D . Quality of life of patients with fibrodysplasia ossificans progressiva. J Child Orthop. 2015;9(6):489‐493. 26564023 10.1007/s11832-015-0704-6 PMC4661157

[4] Connor JM , Evans DA . Fibrodysplasia ossificans progressiva. The clinical features and natural history of 34 patients. J Bone Joint Surg Br. 1982;64(1):76‐83. 7068725 10.1302/0301-620X.64B1.7068725

[5] Kaplan FS , Zasloff MA , Kitterman JA , Shore EM , Hong CC , Rocke DM . Early mortality and cardiorespiratory failure in patients with fibrodysplasia ossificans progressiva. J Bone Joint Surg Am. 2010;92(3):686‐691. 20194327 10.2106/JBJS.I.00705 PMC2827822

[6] Shore EM , Xu M , Feldman GJ , et al. A recurrent mutation in the BMP type I receptor ACVR1 causes inherited and sporadic fibrodysplasia ossificans progressiva. Nat Genet. 2006;38(5):525‐527. 16642017 10.1038/ng1783

[7] Zhang W , Zhang K , Song L , et al. The phenotype and genotype of fibrodysplasia ossificans progressiva in China: a report of 72 cases. Bone. 2013;57(2):386‐391. 24051199 10.1016/j.bone.2013.09.002 PMC3975922

[8] Kaplan FS , Glaser DL , Pignolo RJ , Shore EM . A new era for fibrodysplasia ossificans progressiva: a druggable target for the second skeleton. Expert Opin Biol Ther. 2007;7(5):705‐712. 17477807 10.1517/14712598.7.5.705

[9] Kaplan FS , Al Mukaddam M , Baujat G , et al. The medical management of fibrodysplasia ossificans progressiva: current treatment considerations. Proceedings of the International Clinical Council (ICC) on Fibrodysplasia Ossificans Progressiva (FOP). 2019;1:1‐111.

[10] Kaplan FS , Tabas JA , Gannon FH , Finkel G , Hahn GV , Zasloff MA . The histopathology of fibrodysplasia ossificans progressiva. An endochondral process. J Bone Joint Surg Am. 1993;75(2):220‐230. 7678595 10.2106/00004623-199302000-00009

[11] Pacifici M , Cossu G , Molinaro M , Tato F . Vitamin A inhibits chondrogenesis but not myogenesis. Exp Cell Res. 1980;129(2):469‐474. 7428831 10.1016/0014-4827(80)90517-0

[12] Shimono K , Tung WE , Macolino C , et al. Potent inhibition of heterotopic ossification by nuclear retinoic acid receptor‐gamma agonists. Nat Med. 2011;17(4):454‐460. 21460849 10.1038/nm.2334 PMC3073031

[13] Weston AD , Chandraratna RA , Torchia J , Underhill TM . Requirement for RAR‐mediated gene repression in skeletal progenitor differentiation. J Cell Biol. 2002;158(1):39‐51. 12105181 10.1083/jcb.200112029 PMC2173026

[14] Hind M , Stinchcombe S . Palovarotene, a novel retinoic acid receptor gamma agonist for the treatment of emphysema. Curr Opin Investig Drugs. 2009;10(11):1243‐1250. 19876792

[15] Chakkalakal SA , Uchibe K , Convente MR , et al. Palovarotene inhibits heterotopic ossification and maintains limb mobility and growth in mice with the human ACVR1(R206H) fibrodysplasia ossificans progressiva (FOP) mutation. J Bone Miner Res. 2016;31(9):1666‐1675. 26896819 10.1002/jbmr.2820 PMC4992469

[16] Lindborg CM , Brennan TA , Wang H , Kaplan FS , Pignolo RJ . Cartilage‐derived retinoic acid‐sensitive protein (CD‐RAP): a stage‐specific biomarker of heterotopic endochondral ossification (HEO) in fibrodysplasia ossificans progressiva (FOP). Bone. 2018;109:153‐157. 28963080 10.1016/j.bone.2017.09.016 PMC7680581

[17] Pignolo RJ , Bedford‐Gay C , Liljesthrom M , et al. The natural history of flare‐ups in fibrodysplasia ossificans progressiva (FOP): a comprehensive global assessment. J Bone Miner Res. 2016;31(3):650‐656. 27025942 10.1002/jbmr.2728 PMC4829946

[18] Rajapakse CS , Lindborg C , Wang H , et al. Analog method for radiographic assessment of heterotopic bone in fibrodysplasia ossificans progressiva. Acad Radiol. 2017;24(3):321‐327. 27989444 10.1016/j.acra.2016.10.010 PMC5309155

[19] Hicks CL , von Baeyer CL , Spafford PA , van Korlaar I , Goodenough B . The faces pain scale‐revised: toward a common metric in pediatric pain measurement. Pain. 2001;93(2):173‐183. 11427329 10.1016/S0304-3959(01)00314-1

[20] Mattera MS , Kaplan FS , Pignolo RJ , Grogan D , Revicki DA . Patient‐reported physical function outcome measure for adults with fibrodysplasia ossificans progressiva: intelligent test design based on PROMIS item banks. Value Health. 2015;18(3):A165.

[21] Pignolo RJ , Kimel M , Whalen J , et al. Validity and reliability of the Fibrodysplasia Ossificans Progressiva Physical Function Questionnaire (FOP‐PFQ), a patient‐reported, disease‐specific measure. *J Bone Miner Res*. 2020;35 Suppl 1. [Presented at the Annual Meeting of the American Society for Bone and Mineral Research (ASBMR)]; 2020, September 11‐15; Virtual Event. Available from: https://www.asbmr.org/meetings/annualmeeting/AbstractDetail?aid=9ea775a5-13b9-4b81-9736-0bbc94ad5d41. 10.1016/j.bone.2022.116642 36526263

[22] Pignolo RJ , Baujat G , Brown MA , et al. Natural history of fibrodysplasia ossificans progressiva: cross‐sectional analysis of annotated baseline phenotypes. Orphanet J Rare Dis. 2019;14(1):98. 31053156 10.1186/s13023-019-1068-7 PMC6499994

[23] Forrest CB , Bevans KB , Pratiwadi R , et al. Development of the PROMIS((R)) pediatric global health (PGH‐7) measure. Qual Life Res. 2014;23(4):1221‐1231. 24264804 10.1007/s11136-013-0581-8 PMC3966936

[24] Hays RD , Bjorner JB , Revicki DA , Spritzer KL , Cella D . Development of physical and mental health summary scores from the Patient‐Reported Outcomes Measurement Information System (PROMIS) global items. Qual Life Res. 2009;18(7):873‐880. 19543809 10.1007/s11136-009-9496-9 PMC2724630

[25] Posner K , Brown GK , Stanley B , et al. The Columbia‐Suicide Severity Rating Scale: initial validity and internal consistency findings from three multisite studies with adolescents and adults. Am J Psychiatry. 2011;168(12):1266‐1277. 22193671 10.1176/appi.ajp.2011.10111704 PMC3893686

[26] Char DS , Hutchison HT , Kitterman JA , Gregory GA . General anesthesia treatment of propriospinal myoclonus in a patient with fibrodysplasia ossificans progressiva. A A Case Rep. 2014;3(1):6‐8. 25612266 10.1213/XAA.0000000000000037

[27] Towler OW , Shore EM , Kaplan FS . Skeletal malformations and developmental arthropathy in individuals who have fibrodysplasia ossificans progressiva. Bone. 2020;130:115116. 31655222 10.1016/j.bone.2019.115116

[28] ClinicalTrials.gov . 2014. Identifier: NCT02279095 An open‐label extension study of palovarotene treatment in FOP. Available at: https://clinicaltrials.gov/ct2/show/NCT02279095 [last accessed: 31 July 2022].

[29] Kaplan FS , Al Mukaddam M , Pignolo RJ . A cumulative analogue joint involvement scale (CAJIS) for fibrodysplasia ossificans progressiva (FOP). Bone. 2017;101:123‐128. 28465250 10.1016/j.bone.2017.04.015

[30] Bruno NP , Beacham BE , Burnett JW . Adverse effects of isotretinoin therapy. Cutis. 1984;33(5):484‐486 489. 6236956

[31] Katz HI , Waalen J , Leach EE . Acitretin in psoriasis: an overview of adverse effects. J Am Acad Dermatol. 1999;41(3 Pt 2):S7‐S12. 10459140 10.1016/s0190-9622(99)70359-2

[32] Mills CM , Marks R . Adverse reactions to oral retinoids. An update. Drug Saf. 1993;9(4):280‐290. 8260121 10.2165/00002018-199309040-00006

[33] Kou S , De Cunto C , Baujat G , et al. Patients with ACVR1R206H mutations have an increased prevalence of cardiac conduction abnormalities on electrocardiogram in a natural history study of fibrodysplasia ossificans progressiva. Orphanet J Rare Dis. 2020;15(1):193. 32727600 10.1186/s13023-020-01465-x PMC7389682

[34] Pignolo RJ , Al Mukaddam M , Baujat G , et al. Palovarotene (PVO) for Fibrodysplasia Ossificans Progressiva (FOP): data from the phase III MOVE trial. *J Bone Miner Res*. 2020;35 Suppl 1. [Presented at the Annual Meeting of the American Society for Bone and Mineral Research (ASBMR)]; 2020, September 11‐15; Virtual Event. Available from: https://www.asbmr.org/meetings/annualmeeting/AbstractDetail?aid=eca7bdf2-2067-444d-b591-420f41845889.

[35] Hsiao EC , Di Rocco M , Cali A , et al. Special considerations for clinical trials in fibrodysplasia ossificans progressiva (FOP). Br J Clin Pharmacol. 2019;85(6):1199‐1207. 30281842 10.1111/bcp.13777 PMC6533500

[36] ClinicalTrials.gov . 2017. Identifier: NCT03312634 An efficacy and safety study of palovarotene for the treatment of fibrodysplasia ossificans progressiva. Available at: https://clinicaltrials.gov/ct2/show/NCT03312634 [last accessed: 31 July 2022].

