# Supplementary material for: Palovarotene for Fibrodysplasia Ossificans Progressiva (FOP): Results of a Randomized, Placebo‐Controlled, Double‐Blind Phase 2 Trial
Source: J Bone Miner Res. 2022 Aug 17;37(10):1891–902. doi: 10.1002/jbmr.4655 (PMC9804935; doi:10.1002/jbmr.4655)
Supplement: Supplementary file 1 — Table S1 Weight‐Adjusted Palovarotene Doses Table S2. Schedule of Key Assessments at Baseline and Weeks 6 and 12 Table S3. Heterotopic Ossification Scores (from Rajapakse and colleagues(19)) Table S4. Incidence of Biomarker Readings Above the Upper Limit of Normal or Below the Lower Limit of Normal by Treatment Group Table S5. Power to Detect a Significant Dose Trend Under Various Assumptions of Response Across the Three Treatment Groups Table S6. LSmean Change from Baseline Scores for FOP‐PFQ and PROMIS at Week 12 Table S7. Raw Means and LSmeans for New HO Volume, New HO Area, Pain and Swelling NRS, and Changes from Baseline in FOP‐PFQ and Adult and Pediatric PROMIS at Week 12 Fig. S1. Trial Schematic Fig. S2. Incidence of Flare‐Ups with New HO at the Flare‐Up Body Region at Week 6 and Week 12 as Assessed by CT Scan or Plain Radiograph (Primary Read) Fig. S3: LSmean Area of New HO at the Flare‐Up Body Region in All Flare‐Ups as Assessed by Plain Radiograph (Primary Read) Fig. S4. LSmean Change from Baseline in Pain Numeric Rating Scale at Each Assessed Time Point [file JBMR-37-1891-s001.docx]

# Supplemental Tables

## Table S1. Weight-Adjusted Palovarotene Doses

| **Weight range category** | **10 mg equivalent** | **5 mg equivalent** | **2.5 mg equivalent** |
| --- | --- | --- | --- |
| **20 to <40 kg** | 6 mg | 3 mg | 1.5 mg |
| **40 to <60 kg** | 8 mg | 4 mg | 2 mg |
| **≥60 kg** | 10 mg | 5 mg | 2.5 mg |

## Table S2. Schedule of Key Assessments at Baseline and Weeks 6 and 12

| **Assessment/Procedure** | **Baseline** | **Week 6**  **End of  Treatment  Period** | **Week 12**  **End of Follow-Up Period** |
| --- | --- | --- | --- |
| Radiograph at flare-up body region | X | X | X |
| Low-dose CT scan at flare-up body region | X | X | X |
| MRI or ultrasound (if unable to undergo MRI) at flare-up body region | X | X | X |
| Physical examination | X | X | X |
| NRS or FPS-R^a^ | X | X | X |
| FOP-PFQ | X | X | X |
| PROMIS Global Health Scale | X | X | X |
| Adverse events | X | X | X |
| Knee and hand/wrist radiographs | X |  | X |
| ^a^FPS-R for patients under 8 years of age. CT: computed tomography; FPS-R: Faces Pain Scale-Revised; MRI: magnetic resonance imaging; NRS: numeric rating scale; PFQ: physical function questionnaire; PROMIS: Patient Reported Outcomes Measurement Information System; ROM: range of motion. | | | |

## Table S3. Heterotopic Ossification Scores (from Rajapakse et al (19))

| **Score** | **Feature** |
| --- | --- |
| 0 | No HO |
| 1 | Single or multiple spicules (punctate) or islands (non-contiguous) of HO |
| 2 | Coalescing islands or reticular complexes of bone |
| 3 | Single contiguous HO having longest dimension ≤½ the diameter of the reference normotopic bone in any projection |
| 4 | Single contiguous HO with longest dimension >½ but ≤1 diameter of the reference normotopic bone in any projection |
| 5 | Single contiguous HO with longest dimension >1 but ≤2 diameter of the reference normotopic bone in any projection |
| 6 | Single contiguous HO with longest dimension >2 diameters of the reference normotopic bone in any projection |
| Clinical images corresponding to these scores can be found online at [https://www.ncbi.nlm.nih.gov/pmc/articles/PMC5309155](https://www.ncbi.nlm.nih.gov/pmc/articles/PMC5309155/). HO: heterotopic ossification. | |

## Table S4. Incidence of Biomarker Readings Above the Upper Limit of Normal or Below the Lower Limit of Normal by Treatment Group

| **Biomarker** | **Placebo (N=10)** | **PVO 5/2.5 mg (N=9)** | **PVO 10/5 mg (N=21)** |
| --- | --- | --- | --- |
| **Bone and cartilage** | | | |
| **Osteocalcin** |  |  |  |
| Baseline | n=6 | n=8 | n=19 |
| Low (<LLN) | 0 | 2 (25.0) | 4 (21.1) |
| Normal | 6 (100.0) | 6 (75.0) | 13 (73.7) |
| High (>ULN) | 0 | 0 | 1 (5.3) |
| Week 6 | n=9 | n=8 | n=16 |
| Low (<LLN) | 0 | 0 | 1 (6.3) |
| Normal | 5 (55.6) | 7 (87.5) | 6 (37.5) |
| High (>ULN) | 4 (44.4) | 1 (12.5) | 9 (56.3) |
| Week 12 | n=9 | n=9 | n=18 |
| Low (<LLN) | 0 | 1 (11.1) | 2 (11.1) |
| Normal | 7 (77.8) | 6 (66.7) | 8 (44.4) |
| High (>ULN) | 2 (22.2) | 2 (22.2) | 8 (44.4) |
| **Bone-specific alkaline phosphatase** |  |  |  |
| Baseline | n=7 | n=7 | n=19 |
| Low (<LLN) | 1 (14.3) | 0 | 1 (5.3) |
| Normal | 5 (71.4) | 7 (100.0) | 17 (89.5) |
| High (>ULN) | 1 (14.3) | 0 | 1 (5.3) |
| Week 6 | n=10 | n=9 | n=18 |
| Low (<LLN) | 1 (10.0) | 0 | 3 (16.7) |
| Normal | 8 (80.0) | 9 (100.0) | 14 (77.8) |
| High (>ULN) | 1 (10.0) | 0 | 1 (5.6) |
| Week 12 | n=9 | n=9 | n=17 |
| Low (<LLN) | 1 (11.1) | 1 (11.1) | 0 |
| Normal | 7 (77.8) | 8 (88.9) | 16 (94.1) |
| High (>ULN) | 1 (11.1) | 0 | 1 (5.9) |
| **P1CP-C-terminal propeptide of type 1 procollagen** |  |  |  |
| Baseline | n=8 | n=8 | n=20 |
| Low (<LLN) | 4 (50.0) | 4 (50.0) | 7 (35.0) |
| Normal | 4 (50.0) | 4 (50.0) | 11 (55.0) |
| High (>ULN) | 0 | 0 | 2 (10.0) |
| Week 6 | n=10 | n=9 | n=19 |
| Low (<LLN) | 3 (30.0) | 1 (11.1) | 2 (10.5) |
| Normal | 5 (50.0) | 8 (88.9) | 16 (84.2) |
| High (>ULN) | 2 (20.0) | 0 | 1 (5.3) |
| Week 12 | n=9 | n=9 | n=18 |
| Low (<LLN) | 2 (22.2) | 3 (33.3) | 4 (22.2) |
| Normal | 5 (55.6) | 6 (66.7) | 13 (72.2) |
| High (>ULN) | 2 (22.2) | 0 | 1 (5.6) |
| **P1NP-N-terminal propeptide of type 1 procollagen** |  |  |  |
| Baseline | n=6 | n=3 | n=12 |
| Low (<LLN) | 1 (16.7) | 0 | 3 (25.0) |
| Normal | 5 (83.3) | 3 (100.0) | 8 (66.7) |
| High (>ULN) | 0 | 0 | 1 (8.3) |
| Week 6 | n=6 | n=3 | n=12 |
| Low (<LLN) | 0 | 0 | 3 (25.0) |
| Normal | 4 (66.7) | 2 (66.7) | 5 (41.7) |
| High (>ULN) | 2 (33.3) | 1 (33.3) | 4 (33.3) |
| Week 12 | n=7 | n=3 | n=13 |
| Low (<LLN) | 1 (14.3) | 0 | 3 (23.1) |
| Normal | 4 (57.1) | 2 (66.7) | 5 (38.5) |
| High (>ULN) | 2 (28.6) | 1 (33.3) | 5 (38.5) |
| **CD retinoic acid-sensitive protein** |  |  |  |
| Baseline | n=3 | n=4 | n=12 |
| Low (<LLN) | 0 | 0 | 0 |
| Normal | 3 (100.0) | 4 (100.0) | 11 (91.7) |
| High (>ULN) | 0 | 0 | 1 (8.3) |
| Week 6 | n=4 | n=5 | n=12 |
| Low (<LLN) | 0 | 0 | 0 |
| Normal | 2 (50.0) | 3 (60.0) | 9 (75.0) |
| High (>ULN) | 2 (50.0) | 2 (40.0) | 3 (25.0) |
| Week 12 | n=4 | n=5 | n=12 |
| Low (<LLN) | 0 | 0 | 0 |
| Normal | 3 (75.0) | 3 (60.0) | 11 (91.7) |
| High (>ULN) | 1 (25.0) | 2 (40.0) | 1 (8.3) |
| **CTX** |  |  |  |
| Baseline | n=8 | n=8 | n=21 |
| Low (<LLN) | 1 (12.5) | 0 | 0 |
| Normal | 7 (87.5) | 8 (100.0) | 21 (100.0) |
| High (>ULN) | 0 | 0 | 0 |
| Week 6 | n=10 | n=9 | n=19 |
| Low (<LLN) | 0 | 0 | 0 |
| Normal | 10 (100.0) | 9 (100.0) | 18 (94.7) |
| High (>ULN) | 0 | 0 | 1 (5.3) |
| Week 12 | n=9 | n=9 | n=18 |
| Low (<LLN) | 0 | 0 | 0 |
| Normal | 9 (100.0) | 9 (100.0) | 17 (94.4) |
| High (>ULN) | 0 | 0 | 1 (5.6) |
| **Angiogenesis** | | | |
| **Urinary basic fibroblast growth factor** |  |  |  |
| Baseline | n=9 | n=6 | n=19 |
| Low (<LLN) | 0 | 0 | 0 |
| Normal | 7 (77.8) | 4 (66.7) | 11 (57.9) |
| High (>ULN) | 2 (22.2) | 2 (33.3) | 8 (42.1) |
| Week 6 | n=6 | n=9 | n=20 |
| Low (<LLN) | 0 | 0 | 0 |
| Normal | 4 (66.7) | 9 (100.0) | 18 (90.0) |
| High (>ULN) | 2 (33.3) | 0 | 2 (10.0) |
| Week 12 | n=6 | n=8 | n=20 |
| Low (<LLN) | 0 | 0 | 0 |
| Normal | 5 (83.3) | 6 (75.0) | 17 (85.0) |
| High (>ULN) | 1 (16.7) | 2 (25.0) | 3 (15.0) |
| **Inflammation** | | | |
| **Erythrocyte sedimentation rate** |  |  |  |
| Baseline | n=9 | n=9 | n=17 |
| Low (<LLN) | 0 | 0 | 0 |
| Normal | 9 (100.0) | 9 (100.0) | 15 (88.2) |
| High (>ULN) | 0 | 0 | 2 (11.8) |
| Week 6 | n=8 | n=6 | n=15 |
| Low (<LLN) | 0 | 0 | 0 |
| Normal | 8 (100.0) | 6 (100.0) | 14 (93.3) |
| High (>ULN) | 0 | 0 | 1 (6.7) |
| Week 12 | n=9 | n=8 | n=13 |
| Low (<LLN) | 0 | 0 | 0 |
| Normal | 8 (88.9) | 8 (100.0) | 13 (100.0) |
| High (>ULN) | 1 (11.1) | 0 | 0 |
| **CRP** |  |  |  |
| Baseline | n=7 | n=7 | n=16 |
| Low (<LLN) | 0 | 0 | 0 |
| Normal | 5 (71.4) | 5 (71.4) | 11 (68.8) |
| High (>ULN) | 2 (28.6) | 2 (28.6) | 5 (31.3) |
| Week 6 | n=10 | n=9 | n=19 |
| Low (<LLN) | 0 | 0 | 0 |
| Normal | 7 (70.0) | 7 (77.8) | 14 (73.7) |
| High (>ULN) | 3 (30.0) | 2 (22.2) | 5 (26.3) |
| Week 12 | n=10 | n=9 | n=19 |
| Low (<LLN) | 0 | 0 | 0 |
| Normal | 9 (90.0) | 5 (55.6) | 15 (78.9) |
| High (>ULN) | 1 (10.0) | 4 (44.4) | 4 (21.1) |
| **IL-6** |  |  |  |
| Baseline | n=8 | n=8 | n=20 |
| Low (<LLN) | 0 | 1 (12.5) | 2 (10.0) |
| Normal | 8 (100.0) | 7 (87.5) | 18 (90.0) |
| High (>ULN) | 0 | 0 | 0 |
| Week 6 | n=10 | n=9 | n=19 |
| Low (<LLN) | 0 | 0 | 0 |
| Normal | 10 (100.0) | 9 (100.0) | 19 (100.0) |
| High (>ULN) | 0 | 0 | 0 |
| Week 12 | n=9 | n=9 | n=19 |
| Low (<LLN) | 0 | 0 | 3 (15.8) |
| Normal | 9 (100.0) | 9 (100.0) | 16 (84.2) |
| High (>ULN) | 0 | 0 | 0 |
| **IL-1 beta** |  |  |  |
| Baseline | n=8 | n=8 | n=20 |
| Low (<LLN) | 0 | 0 | 0 |
| Normal | 2 (25.0) | 3 (37.5) | 12 (60.0) |
| High (>ULN) | 6 (75.0) | 5 (62.5) | 8 (40.0) |
| Week 6 | n=10 | n=9 | n=19 |
| Low (<LLN) | 0 | 0 | 0 |
| Normal | 4 (40.0) | 3 (33.3) | 12 (63.2) |
| High (>ULN) | 6 (60.0) | 6 (66.7) | 7 (36.8) |
| Week 12 | n=9 | n=9 | n=19 |
| Low (<LLN) | 0 | 0 | 0 |
| Normal | 4 (44.4) | 3 (33.3) | 12 (63.2) |
| High (>ULN) | 5 (55.6) | 6 (66.7) | 7 (36.8) |
| **TNF-alpha** |  |  |  |
| Baseline | n=8 | n=8 | n=20 |
| Low (<LLN) | 4 (50.0) | 4 (50.0) | 7 (35.0) |
| Normal | 4 (50.0) | 4 (50.0) | 13 (65.0) |
| High (>ULN) | 0 | 0 | 0 |
| Week 6 | n=10 | n=9 | n=19 |
| Low (<LLN) | 3 (30.0) | 3 (33.3) | 0 |
| Normal | 7 (70.0) | 6 (66.7) | 19 (100.0) |
| High (>ULN) | 0 | 0 | 0 |
| Week 12 | n=9 | n=9 | n=19 |
| Low (<LLN) | 2 (22.2) | 2 (22.2) | 3 (15.8) |
| Normal | 7 (77.8) | 7 (77.8) | 16 (84.2) |
| High (>ULN) | 0 | 0 | 0 |
| **Creatine phosphokinase** |  |  |  |
| Baseline | n=6 | n=8 | n=17 |
| Low (<LLN) | 0 | 1 (12.5) | 0 |
| Normal | 6 (100.0) | 7 (87.5) | 17 (100.0) |
| High (>ULN) | 0 | 0 | 0 |
| Week 6 | n=10 | n=9 | n=19 |
| Low (<LLN) | 0 | 1 (11.1) | 0 |
| Normal | 10 (100.0) | 8 (88.9) | 19 (100.0) |
| High (>ULN) | 0 | 0 | 0 |
| Week 12 | n=10 | n=9 | n=19 |
| Low (<LLN) | 0 | 0 | 1 (5.3) |
| Normal | 9 (90.0) | 9 (100.0) | 18 (94.7) |
| High (>ULN) | 1 (10.0) | 0 | 0 |
| **Lactate dehydrogenase** |  |  |  |
| Baseline | n=7 | n=8 | n=20 |
| Low (<LLN) | 0 | 1 (12.5) | 0 |
| Normal | 7 (100.0) | 7 (87.5) | 17 (85.0) |
| High (>ULN) | 0 | 0 | 3 (15.0) |
| Week 6 | n=10 | n=9 | n=16 |
| Low (<LLN) | 0 | 0 | 0 |
| Normal | 9 (90.0) | 8 (88.9) | 13 (81.3) |
| High (>ULN) | 1 (10.0) | 1 (11.1) | 3 (18.8) |
| Week 12 | n=10 | n=9 | n=18 |
| Low (<LLN) | 0 | 0 | 1 (5.6) |
| Normal | 10 (100.0) | 9 (100.0) | 16 (88.9) |
| High (>ULN) | 0 | 0 | 1 (5.6) |

CD: cartilage-derived; CRP: C-reactive protein; CTX: C-terminal telopeptide; IL: interleukin; LLN: lower limit of normal; PVO: palovarotene; TNF: tumor necrosis factor; ULN: upper limit of normal.

## Table S5. Power to Detect a Significant Dose Trend Under Various Assumptions of Response Across the Three Treatment Groups

|  | **Patients with no or minimal new HO, n (%)** | | |  |
| --- | --- | --- | --- | --- |
| **Scenario** | **Placebo  (N=8)** | **PVO 5/2.5 mg (N=6)** | **PVO 10/5 mg (N=18)** | **Power, %** |
| **1** | 2/10 (20.0) | 2/9 (22.2) | 13/21 (61.9) | 81.0 |
| **2** | 2/10 (20.0) | 4/9 (44.4) | 15/21 (71.4) | 90.1 |
| **3** | 2/10 (20.0) | 6/9 (66.7) | 17/21 (81.0) | 96.8 |
| **4** | 3/10 (30.0) | 3/9 (33.3) | 13/21 (61.9) | 57.0 |
| **5** | 3/10 (30.0) | 4/9 (44.4) | 15/21 (71.4) | 75.0 |
| **6** | 3/10 (30.0) | 6/9 (66.7) | 17/21 (81.0) | 88.1 |

Power calculations were based on predicted enrollment. The actual number of patients enrolled was greater, increasing the power. HO: heterotopic ossification; PVO: palovarotene.

## Table S6. LSmean Change from Baseline Scores for FOP-PFQ and PROMIS at Week 12

|  | **Placebo (N=10)** | | **PVO 5/2.5 mg (N=9)** | **PVO 10/5 mg (N=21)** | |
| --- | --- | --- | --- | --- | --- |
| **FOP-PFQ^a^** |  | |  |  | |
| Baseline, mean (SD) | 52.6 (19.5) | | 51.7 (20.3) | 49.3 (26.5) | |
| Change at week 12, LSmean (SE) | 3.0 (2.4) | | 1.1 (2.6) | 4.1 (1.7) | |
| **PROMIS Global Health Scale T-scores, adults^b^** |  | |  |  | |
| Number of adult patients | 5 | | 5 | 17 | |
| Baseline Global Physical Health, mean (SD) | 37.8 (6.8) | | 43.6 (6.1) | 41.0 (9.2) | |
| Change at week 12, LSmean (SE) | 3.0 (2.3) | | 5.6 (2.3) | 4.4 (1.3) | |
| Baseline Global Mental Health, mean (SD) | 45.9 (3.8) | | 51.0 (6.3) | 51.2 (6.9) | |
| Change at week 12, LSmean (SE) | 0.6 (2.7) | | 3.2 (2.6) | 4.6 (1.5) | |
| **PROMIS Global Health Scale T-scores, pediatric^c^** | |  | | |  |
| Number of pediatric patients | 5 | | 4 | 4 | |
| Baseline, mean (SD) | 39.4 (5.7) | | 42.1 (8.4) | 35.7 (0.9) | |
| Change at week 12, LSmean (SE) | -6.6 (2.6) | | -2.5 (2.3) | 0.7 (2.7) | |
| Full analysis set. ^a^Percentage of worst total score – higher scores indicate worse physical function; ^b^Adult questionnaires were completed for patients 15 years of age and older – higher scores indicate better global health; ^c^Pediatric PROMIS has only a total score (no subscores) – higher scores indicate better global health. FOP-PFQ: fibrodysplasia ossificans progressiva physical function questionnaire; LS: least squares; PROMIS: Patient Reported Outcomes Measurement Information System; PVO: palovarotene; SD: standard deviation; SE: standard error. | | | | | |

## Table S7. Raw Means and LSmeans for New HO Volume, New HO Area, Pain and Swelling NRS, and Changes from Baseline in FOP-PFQ and Adult and Pediatric PROMIS at Week 12

| **Outcome** |  | **Group** |  |
| --- | --- | --- | --- |
| **New HO Volume at Week 12, x10^3^ mm^3^** | **Placebo (N=10)** | **PVO 5/2.5 mg (N=9)** | **PVO 10/5 mg (N=20)^a^** |
| Mean (SD) | 16.2 (41.6) | 1.2 (3.2) | 3.9 (11.9) |
| LSmean (SE) | 18.0 (7.1) | 1.3 (7.5) | 3.8 (5.0) |
| **New HO Volume at Week 12 in Flare-Ups with New HO, x10^3^ mm^3^** | **Placebo (N=3)** | **PVO 5/2.5 mg (N=2)** | **PVO 10/5 mg (N=4)** |
| Mean (SD) | 53.9 (68.9) | 5.3 (6.1) | 16.4 (21.8) |
| LSmean (SE) | 58.8 (22.5) | 10.3 (27.4) | 10.3 (20.0) |
| **New HO Area at Week 12, mm^2^** | **Placebo (N=10)** | **PVO 5/2.5 mg (N=9)** | **PVO 10/5 mg (N=20)** |
| Mean (SD) | 621.7 (1287.5) | 71.2 (213.7) | 19.0 (85.0) |
| LSmean (SE) | 789.5 (184.0) | 86.0 (171.9) | 25.1 (121.4) |
| **Change from Baseline to Week 12 in Pain NRS** | **Placebo (N=10)** | **PVO 5/2.5 mg (N=9)** | **PVO 10/5 mg (N=20)** |
| Mean (SD) | -2.2 (2.5) | -1.9 (2.4) | -3.6 (2.7) |
| LSmean (SE) | -2.1 (0.7) | -2.6 (0.7) | -3.3 (0.5) |
| **Change from Baseline to Week 12 in  Swelling NRS** | **Placebo (N=10)** | **PVO 5/2.5 mg (N=9)^b^** | **PVO 10/5 mg (N=20)** |
| Mean (SD) | -2.3 (3.2) | -2.5 (2.2) | -2.1 (3.7) |
| LSmean (SE) | -2.1 (0.7) | -2.3 (0.7) | -2.2 (0.5) |
| **Change from Baseline to Week 12 in  FOP-PFQ** | **Placebo (N=10)** | **PVO 5/2.5 mg (N=9)** | **PVO 10/5 mg (N=21)^c^** |
| Mean (SD) | 3.0 (9.6) | 1.1 (8.7) | 4.2 (7.9) |
| LSmean (SE) | 3.0 (2.4) | 1.1 (2.6) | 4.1 (1.7) |
| **Change from Baseline to Week 12 in PROMIS Global Health Scale T-scores, adults** | **Placebo (N=5)** | **PVO 5/2.5 mg (N=5)** | **PVO 10/5 mg (N=17)^d^** |
| Mean (SD) Global Physical Health | 3.8 (3.9) | 5.0 (6.2) | 4.0 (6.8) |
| LSmean (SE) Global Physical Health | 3.0 (2.3) | 5.6 (2.3) | 4.4 (1.3) |
| Mean (SD) Global Mental Health | 1.0 (3.7) | 3.1 (4.1) | 4.3 (7.5) |
| LSmean (SE) Global Mental Health | 0.6 (2.7) | 3.2 (2.6) | 4.6 (1.5) |
| **Change from Baseline to Week 12 in PROMIS Global Health Scale T-scores, pediatric** | **Placebo (N=5)^e^** | **PVO 5/2.5 mg (N=4)** | **PVO 10/5 mg (N=4)^e^** |
| Mean (SD) Global Health | -6.6 (2.0) | -2.1 (3.0) | 0.2 (7.6) |
| LSmean (SE) Global Health | -6.6 (2.6) | -2.5 (2.3) | 0.7 (2.7) |

Analysis set is per protocol set for all outcomes except for FOP-PFQ and PROMIS scores, which are reported for the full analysis set. ^a^N=17 for mean; ^b^N=8 for mean; ^c^N=20 for mean; ^d^N=16 for mean; ^e^N=3 for mean. FOP-PFQ: fibrodysplasia ossificans progressiva physical function questionnaire; HO: heterotopic ossification; LS: least squares; NRS: numeric rating scale; PROMIS: Patient Reported Outcomes Measurement Information System; PVO: palovarotene; SD: standard deviation; SE: standard error.

# Supplemental Figures

##
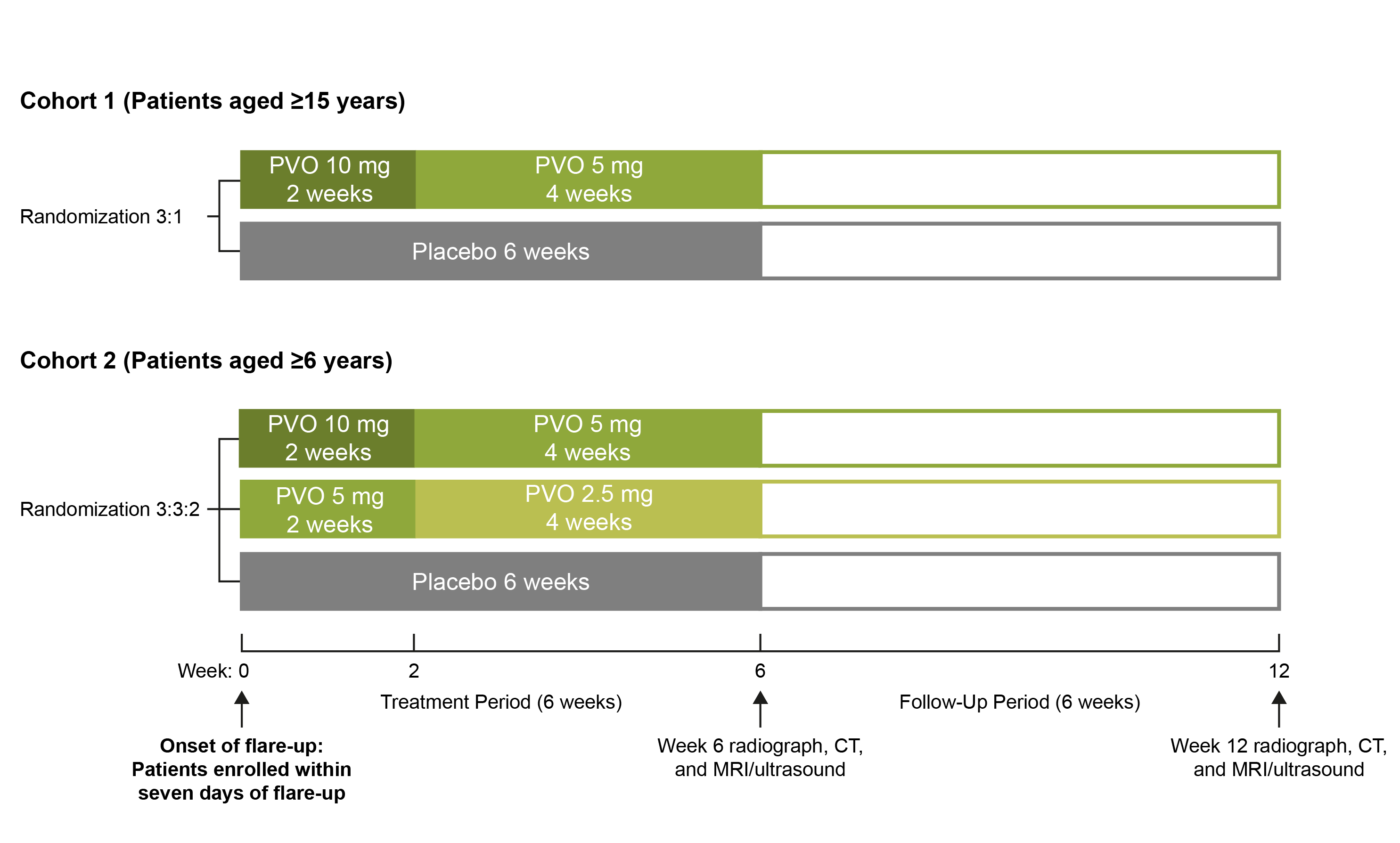
Figure S1. Trial Schematic

CT: computed tomography; HO: heterotopic ossification; MRI: magnetic resonance imaging; PVO: palovarotene.

## Figure S2. Incidence of Flare-Ups with New HO at the Flare-Up Body Region at Week 6 and Week 12 as Assessed by CT Scan or Plain Radiograph (Primary Read)

1.
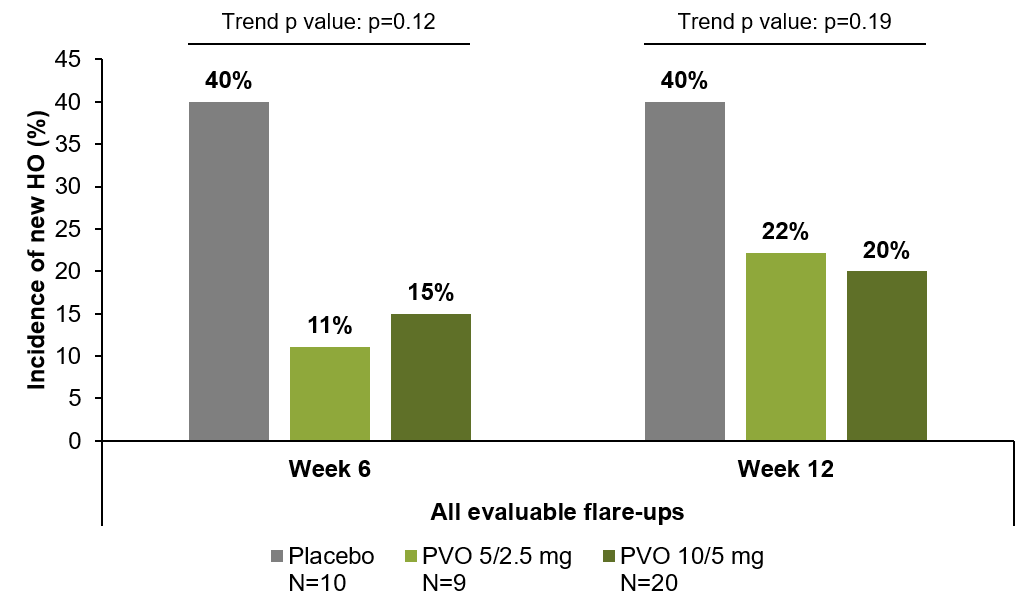
***All Evaluable Flare-Ups***
2.
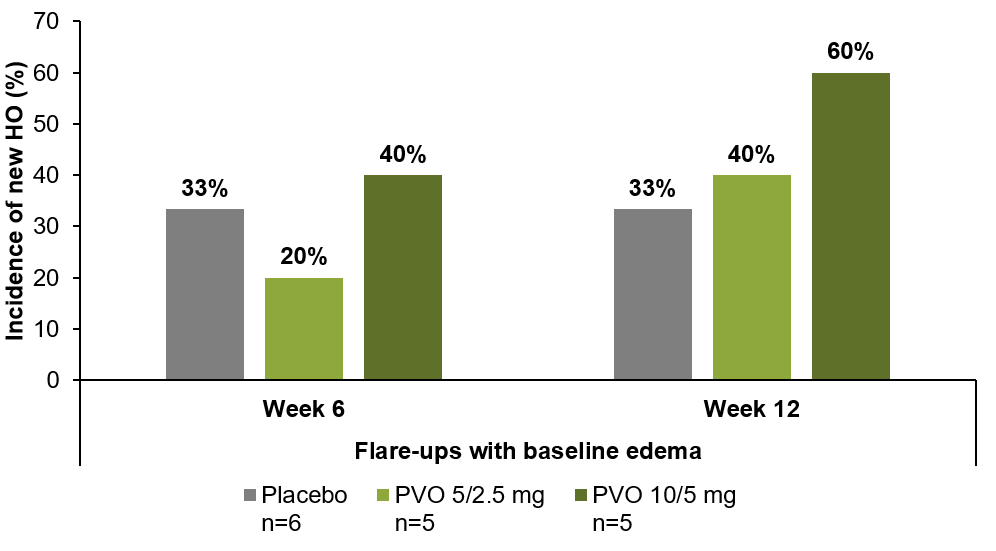
***Flare-Ups with Baseline Edema***

Per protocol analysis set. In the primary read, each imaging modality was assessed independently for HO and/or soft tissue edema. A) Primary analysis: one-sided Cochran-Armitage test of trend for all evaluable flare-ups at week 6 and week 12. CT: computed tomography; HO: heterotopic ossification; PVO: palovarotene.

##
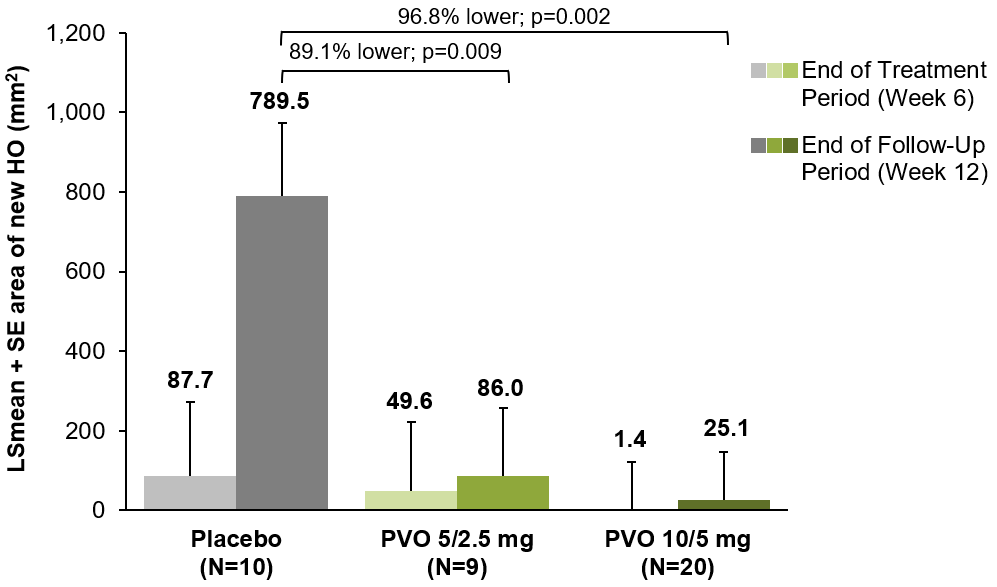
Figure S3: LSmean Area of New HO at the Flare-Up Body Region in All Flare-Ups as Assessed by Plain Radiograph (Primary Read)

Per protocol analysis set. In the primary read, each imaging modality was assessed independently for HO. p values are from pairwise tests from a repeated measures mixed model. Flare-up body regions: placebo: 2 hip, 1 knee; PVO 5/2.5 mg: 1 shoulder/elbow, 1 knee; PVO 10/5 mg: 3 hip, 1 knee. CT: computed tomography; HO: heterotopic ossification; LSmean: least squares mean; PVO: palovarotene; SE: standard error.

## Figure S4. LSmean Change from Baseline in Pain Numeric Rating Scale at Each Assessed Time Point


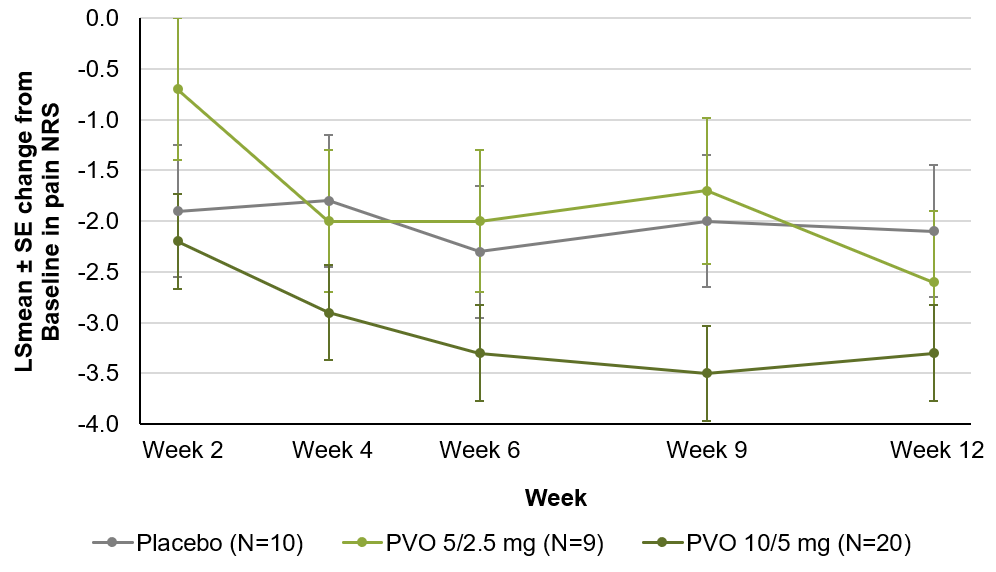
 Per protocol analysis set. LSmean change from baseline in pain NRS rated from 0 (no pain) to 10 (worse pain ever experienced). For children less than 8 years old, pain was rated using the Faces Pain Scale – Revised rated from 0 (no pain) to 10 (very much pain). LSmean: least square means; NRS: numeric rating scale; PVO: palovarotene; SE: standard error.
